# Silica-based optical chemosensors for rapid and reliable on-site detection of gamma-hydroxybutyrate in beverages and oral fluids

**DOI:** 10.1007/s00604-025-07134-9

**Published:** 2025-04-02

**Authors:** Jordi Hernández-Contreras, Jordi Roig-Rubio, Margarita Parra, Salvador Gil, Pau Arroyo, José A. Sáez, Carlos Lodeiro, Pablo Gaviña

**Affiliations:** 1 Instituto Interuniversitario de Investigación de Reconocimiento Molecular y Desarrollo Tecnológico (IDM), Universitat de València - Universitat Politècnica de València, Doctor Moliner 50, Burjassot, 46100 Valencia, Spain; 2 CIBER de Bioingeniería, Biomateriales y Nanomedicina (CIBER-BBN), Madrid, Spain; 3 BIOSCOPE Research Group, LAQV-REQUIMTE, Chemistry Department, NOVA School of Science and Technology, FCT NOVA, Universidade NOVA de Lisboa, 2829-516 Caparica, Portugal; 4 PROTEOMASS Scientific Society, 2825-466 Costa de Caparica, Portugal

**Keywords:** Chemical submission, Silica nanoparticles, Chromo-fluorogenic chemosensors, Gamma-hydroxybutyrate (GHB), Oral fluids, Spiked drinks

## Abstract

The illicit use of gamma-hydroxybutyrate (GHB) in drug-facilitated crimes underscores the urgent need for rapid and reliable detection technologies. This study introduces two innovative silica-based nanosensors that offer real-time, on-site detection of GHB in spiked beverages at concentrations typical of chemical submission cases. Both chemosensors are based on silica nanoparticles functionalized with 2-aminonaphtoxazole derivatives. The first nanosensor, modified with a *p*-nitroaniline chromogenic unit, enables a visible color change for immediate visual identification, while the second incorporates a fluorescein derivative for high-sensitivity detection via fluorescence. Both nanosystems demonstrate exceptional specificity to GHB, with minimal interference from other substances. Moreover, the solid-phase integration of these nanosensors onto hydrophobic cellulose-based substrates enhances their portability and user-friendliness, making them suitable for nightlife venues and forensic applications. Finally, chromogenic precursor **1** has proven to be an excellent chemosensor for the visual detection of GHB in oral fluid, with a calculated LOD of 19.2 μM, and a linear response in the 32–132 μM range.

**Graphical abstract:**

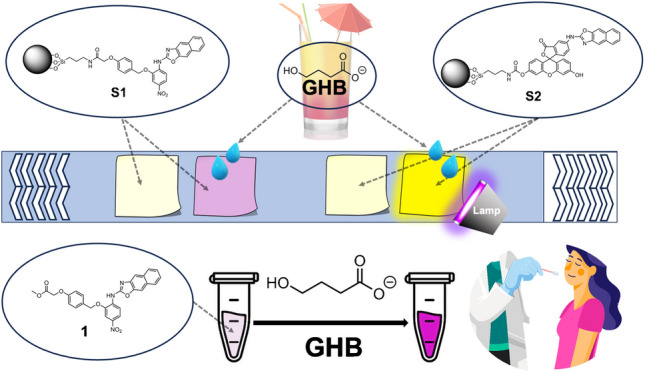

**Supplementary Information:**

The online version contains supplementary material available at 10.1007/s00604-025-07134-9.

## Introduction

Gamma-hydroxybutyrate (GHB) is a short-acting central nervous system depressant, that has garnered significant attention due to its misuse in drug-facilitated crimes, particularly sexual assaults, because of its sedative effects and rapid metabolism, being detected only within 6 h in the blood and 12 h in urine after a single exposure to 55 mg/kg of GHB [1]. The illicit use of GHB is on the rise, with statistics showing an alarming trend in its involvement in such crimes [1–4]. Additionally, GHB is used recreationally, which raises further concerns. As noted in the 2023 European Drug Report, there is an increasing concern about its consumption in recent years, particularly because individuals using mixtures of drugs may be unaware of the substances they are consuming. This can lead to dangerous drug interactions and elevated health risks. For instance, GHB was present in 11% of acute drug toxicity presentations and accounted for 27% of critical care admissions, highlighting the significant risk of overdose [5].

Therefore, this growing concern emphasizes the necessity for rapid and reliable detection of GHB, underscored by its prevalence in nightlife settings and its association with criminal activities. For instance, a report by the European Monitoring Centre for Drugs and Drug Addiction (EMCDDA) indicated that GHB was the fourth most common drug reported by hospitals in 2022 [6]. The increasing use of GHB in sexual assaults contexts demands advanced detection technologies that can provide immediate and accurate results to prevent and address these crimes. Current detection methods, such as gas chromatography-mass spectrometry (GC–MS) and liquid chromatography-mass spectrometry (LC–MS), are highly accurate but often impractical for on-site testing due to their complexity and the time required for analysis [7]. This gap in rapid detection capability can hinder timely intervention and investigation.

An optical response device that can be utilized rapidly and easily by untrained individuals is particularly attractive for quick on-site testing before consuming a suspicious drink or to perform a preliminary forensic triage on oral fluid after a possible consumption of spiked beverages. Unfortunately, the availability of selective and sensitive colorimetric and/or fluorimetric chemosensors for the visual detection of GHB in beverages is still quite limited [8–12]. Some of these sensors have certain drawbacks such as the necessity for organic solvents and high detection limits in aqueous solutions. We have recently reported two 2-aminonaphthoxazole-based chemosensors for the chromogenic and fluorescent detection of GHB in beverages [12]. In both cases, the limits of detection for GHB are much lower than the usual intake found in recreational environments. Considering that these systems were designed to be used in solution, the logical step to increase their usability and portability was their integration and optimization in solid phase devices [13, 14]. On the other hand, the detection of GHB in the saliva of victims of chemical submission is crucial both in forensic investigations and for the protection of affected individuals. The availability of tools to perform a rapid analysis may provide key evidence for potential legal proceedings, helping to identify involuntary substance consumption and supporting cases of assault or abuse. Additionally, its rapid identification in a forensic triage context can expedite medical attention, reduce health risks for the victim, and activate appropriate protection and support mechanisms. The easy availability of saliva from the potential victim for subsequent analysis prompted us to consider the usefulness of a rapid, on-site test for the presence of GHB in this fluid.

**Chart 1:**
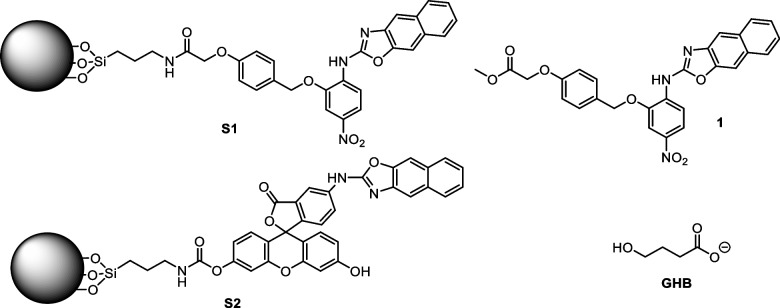
Structure of nanosensors **S1** and **S2**, based on silica nanoparticles functionalized with 2-aminonaphthoxazole recognition units attached to a chromophore (S1) or fluorophore (S2), and chemical structures of chemosensor **1** and GHB

Consequently, in this work, our research focuses primarily on the development of novel nanosensors based on functionalized silica nanoparticles for GHB recognition in complex matrices. These structures have been designed to detect GHB with high sensitivity and specificity through chromogenic and fluorescent changes. The first nanosensor (**S1**) includes a naphthoxazole group with a nitro (NO₂) substituent into its structure, leading to a visible color change when GHB is present in beverages. This color change is particularly useful for quick visual inspection, making it suitable for environments such as bars and clubs where immediate detection is critical. The second nanosensor (**S2**) incorporates a fluorescein group, which emits a fluorescent signal upon interaction with GHB. The fluorescence change offers enhanced sensitivity, capable of detecting low concentrations of GHB, making it ideal not only for clubs, but also for more controlled environments such as laboratories and medical settings. In addition, we have demonstrated that the nitroaromatic containing 2-aminonaphthoxazole precursor **1** in solution is able to detect the presence of GHB in oral fluid, by appearance of a pinkish red color, visible to the naked eye, at concentrations characteristics of exogenous intake of GHB (see Chart 1).

## Experimental section

### Chemicals and instrumentation

All reagents employed in the synthesis were acquired from Sigma Aldrich (Madrid, Spain) and used without further purification. The drugs tested, sodium γ-hydroxybutyrate (GHB), (-)-scopolamine hydrobromide trihydrate, ketamine, methcathinone (ephedrone) and diazepam were purchased by Sigma Aldrich with the required prior authorization of the Agencia Española de Medicamento y Productos Sanitarios (AEMPS). All solvents were ACS reagent grade or better quality and were used without further purification. The solvents such as DMSO and tetrahydrofuran (THF) were purchased from Scharlab S.L. (Barcelona, Spain). ^1^H NMR and ^13^C NMR spectra were registered with Bruker Advance 300 MHz spectrometers, all of them referenced to the corresponding residual solvent peak. High-resolution mass spectra (HRMS) were recorded in the positive ion mode on an AB SCIEX TripleTOF™ 6600 + liquid chromatography/mass spectrometry spectrometer. All photophysical analyses were carried out in air-equilibrated DMSO at 298 K, unless otherwise specified. UV–vis absorption spectra were recorded with a PerkinElmer λ40 or Shimadzu UV-2600 spectrophotometers using quartz cells with a path length of 1.0 cm. Plot2 was the program used to depict titrations. TGA analyses were carried out on TGA/SDTA 851e balance (Mettler Toledo, Columbus, OH, USA) in an oxidizing atmosphere (air, 80 mL min^−1^) with a heating rate program between 393–1273 °C at 10 °C min^−1^, followed by an isothermal heating step at 1273 °C for 30 min. DLS experiments were performed using a ZetaSizer Nano ZS (Malvern, Germany). Images of transmission electron microscopy were taken with JEOL-1010 transmission electron microscopy operating at 100 kV. Silylated paper was obtained from chromatography paper Whatman™, 3mm chr. Real saliva samples were obtained from some of the authors of this work.

### Synthesis of chemosensor 1 and nanosensor S1

#### Synthesis of methyl 2-(4-(hydroxymethyl)phenoxy)acetate (3)

4-(Hydroxymethyl)phenol (**2**) (2.48 g, 20 mmol) and potassium iodide (332 mg, 2 mmol) were dissolved in presence of K_2_CO_3_ (5.52 g, 40 mmol) in 40 mL of acetone. After 1 h stirring at reflux temperature, methyl 2-bromoacetate (2.2 mL, 24 mmol) was added. The reaction mixture was kept under stirring at reflux for 4.5 h. Finally, the solvent was removed, and the product was isolated after a washing and extraction step with AcOEt, as a pale-yellow powder (3.4 g, 87% yield) without further purification. ^1^H NMR (300 MHz, DMSO-*d*_*6*_) δ (ppm): 7.75 – 7.72 (d, 2H), 7.40 – 7.37 (d, 2H), 5.57 (t, 1H), 5.28 (s, 2H), 4.94 – 4.92 (d, 2H) and 4.20 (s, 3H).

#### Synthesis of methyl 2-(4-(bromomethyl)phenoxy)acetate (4)

Methyl 2-(4-(hydroxymethyl)phenoxy)acetate (**3**) (200 mg, 1 mmol) was dissolved in 2 mL of dichloromethane (DCM). Then, a solution of phosphorus tribromide (120 μL, 1.2 mmol) in 2 mL of Et_2_O was prepared and added dropwise to the mixture. The reaction mixture was stirred at room temperature for 30 min. After that, the reaction was quenched by adding 5 mL of H_2_O and the product was extracted with AcOEt. Finally, the solvent was removed, and the final product was isolated as a yellow powder (200 mg, 77% yield) without further purification. ^1^H NMR (300 MHz, CDCl_3_) δ (ppm): 7.35 – 7.32 (m, 2H), 6.87 – 6.85 (m, 2H), 4.64 (s, 2H), 4.48 (s, 2H) and 3.81 (s, 3H). ^13^C NMR (126 MHz, CDCl_3_) δ (ppm): 169.32, 157.93, 131.32, 130.69, 115.05, 65.44, 52.48 and 33.61.

#### Synthesis of methyl 2-(4-((2-amino-5-nitrophenoxy)methyl)phenoxy)acetate (5)

2-Amino-5-nitrophenol (198.8 mg, 1.29 mmol), potassium iodide (21.4 mg, 0.129 mmol) and K_2_CO_3_ (354.4 mg, 2.57 mmol) were dissolved in acetone (12.5 mL). After stirring 30 min at reflux, benzy bromide derivative **4** (400 mg, 1.54 mmol) was added, and the reaction mixture was refluxed for further 4.5 h. Then, the reaction was quenched by adding 15 mL of H_2_O and the product was extracted with AcOEt and treated with anhydrous MgSO_4_. The solvent was removed, and the product was isolated as an orange powder (414.5 mg, 97% yield) without further purification. ^1^H NMR (300 MHz, CDCl_3_) δ (ppm): 7.84 – 7.81 (dd, 1H), 7.77 (d, 1 H), 7.39 – 7.37 (m, 2H), 6.97 – 6.93 (m, 2H), 6.66 – 6.63 (d, 1H), 5.08 (s, 2H), 4.67 (s, 2H) and 3.82 (s, 3H). ^13^C NMR (126 MHz, CDCl_3_) δ (ppm): 169.38, 158.15, 144.65, 143.59, 129.90, 129.16, 119.51, 115.02, 112.09, 107.41. 71.33, 65.45 and 52.48.

#### Synthesis of isothiocyanate 6

Compound **5** (300 mg, 0.903 mmol) was dissolved in 10 mL of DCM. Then, CSCl_2_ (76 μL, 0.903 mmol) was added dropwise to the reaction mixture at 0 ºC, using an ice bath. A saturated aqueous NaHCO_3_ solution (8 mL) was then added, and the mixture was stirred for 7 h at room temperature. Finally, the product was extracted with AcOEt and dried with anhydrous MgSO_4_. After removing the solvent, the product was isolated as a maroon red powder (290.6 mg, 86% yield) without further purification. ^1^H NMR (300 MHz, CDCl_3_) δ (ppm): 7.87 – 7.83 (dd, 1H), 7.80 (d, 1H), 7.45 – 7.43 (m, 2H), 7.19 – 7.17 (d, 1H), 6.96 – 6.93 (m, 2H), 5.20 (s, 2H), 4.66 (s, 2H) and 3.82 (s, 3H). ^13^C NMR (126 MHz, CDCl_3_) δ (ppm): 158.21, 129.57, 124.99, 116.83, 115.03, 107.87, 71.32, 65.46 and 52.48. FTIR-ATR spectrum showed a band located at 2029.5 cm^−1^, which belongs to the isothiocyanate (-SCN) absorbance band.

#### Synthesis of chemosensor 1

3-Amino-2-naphthol (**7**) (102.8 mg, 0.65 mmol) was mixed with thiocyanate **6** (290 mg, 0.75 mmol), in 7 mL of pyridine under argon atmosphere, and the mixture was stirred for 17 h at room temperature. Then, tetrabutylammonium iodide (2.78 mg, 0.0075 mmol) and H_2_O_2_ 30% (170 μL, 1.5 mmol) were added, and the mixture was kept stirring for 5 h at room temperature (the solution turned to copper brown colour immediately after the addition). After that, the reaction mixture was quenched by the addition of 10 mL of a saturated aqueous solution of NH_4_Cl and the product was extracted with AcOEt. Once the solvent was completely removed under vacuum, the product was isolated as a yellow powder (284 mg, 88% yield) without further purification. ^1^H NMR (300 MHz, CDCl_3_) δ (ppm): 8.79 – 8.77 (d, 1H), 8.09 – 8.07 (dd, 1H), 7.94 (s, 2H), 7.92 (d, 1H), 7.73 (s, 1H), 7.46 (m, 2H), 7.44 – 7.42 (d, 2H), 7.01 – 6.99 (d, 2H), 5.21 (s, 2H), 4.70 (s, 2H) and 3.84 (s, 3H). ^13^C NMR (126 MHz, CDCl_3_) δ (ppm): 169.31, 158.56, 146.08, 142.20, 135.54, 133.54, 130.31, 127.91, 124.97, 118.45, 116.77, 115.29, 114.80, 106.75, 105.39, 71.56, 65.44 and 52.53. HRMS: *m/z* calcd for C_27_H_22_N_3_O_7_ [MH]^+^: 500.1458; found 500.1458.

#### Synthesis of nanosensor S1

A mixture of LUDOX® silica nanoparticles functionalized with APTES (**LUDOX®-APTES**) (35 mg,) and compound **1** (50 mg, 0.1 mmol) was suspended in 2 mL of anhydrous THF previously purged with Argon. The suspension was stirred at room temperature for 4 h, and the solid was isolated by centrifugation, washed thoroughly with THF and H_2_O and centrifugated after each wash. Finally, the product was dried overnight at 40 °C (white-yellow solid, 28 mg) (zeta potential and DLS values are collected in the Supporting information).

### Synthesis of nanosensor S2

#### Synthesis of compound 9

3-amino-2-naphthol (**7**) (102 mg, 0.64 mmol) was mixed with fluorescein-5-isothiocyanate **8** (250 mg, 0.64 mmol) in THF (10 mL). Once dissolved, NEt_3_ (112 μL, 0.81 mmol) was added and the mixture was kept stirring overnight at room temperature (solution turned to orange). Next day, tetrabutylammonium iodide (3 mg, 0.0064 mmol) and H_2_O_2_ 30% (86 μL, 1.28 mmol) were added, and the mixture was kept stirring for 4 h more at room temperature (immediately solution turned to brown). Finally, the solvent was removed, and the products were isolated as a maroon powder without further purification (285 mg, 87% yield). ^1^H NMR (300 MHz, DMSO-d6) δ (ppm): 11.17 (s, 1H), 10.11 (s, 2H), 8.52 (d, J = 1.7 Hz, 1H), 7.97 (dd, J_1_ = 8.3 Hz, J_2_ = 1.8 Hz, 1H), 7.59 (d, J = 7.6 Hz, 1H), 7.57 (d, J = 7.6 Hz, 1H), 7.29 (d, J = 8.2 Hz, 1H), 7.28 (t, J = 7.6 Hz, 1H), 7.20 (dd, J_1_ = 8 Hz, J_2_ = 1 Hz, 1H), 6.69 (d, J = 2.3 Hz, 2H), 6.64 (d, J = 8.8 Hz, 2H) and 6.57 (dd, J_1_ = 8.8 Hz, J_2_ = 2.3 Hz, 2H). ^13^C NMR (126 MHz, DMSO-*d*_*6*_) δ (ppm): 169.20, 159.94, 158.04, 152.40, 147.48, 146.23, 142.50, 140.92, 129.61, 127.83, 125.56, 125.16, 124.71, 122.70, 117.51, 113.06, 112.00, 110.25, 109.69, 102.68 and 83.64. HRMS: *m/z* calcd for C_31_H_19_N_2_O_6_ [MH]^+^: 515.1243; found: 515.1239.

#### Synthesis of chemosensor S2

LUDOX® silica nanoparticles functionalized with an isocyanate group (SiNPs-NCO) (200 mg,) and compound **9** (300 mg, 0.6 mmol) were suspended in 7 mL of anhydrous CH_3_CN in a round bottom flask previously purged with Argon. The suspension was stirred at room temperature for 4 h, and the resulting solid was isolated by centrifugation, through CH_3_CN and H_2_O washes. Finally, the product (164 mg) was dried overnight at 40 °C. (zeta potential and DLS values are collected in the Supporting Information).

### Sensing experiments with S1 and S2 in solution

#### UV–VIS and fluorescence titration studies with nanosensors S1 and S2 and GHB in aqueous solution

For nanosensor **S1**, in a 3 mL quartz cell (1.0 cm of path length), 2800 μL of **S1** (0.33 mg/mL in DMSO) was mixed with increasing quantities of a 0.6 mM aqueous solution of GHB until arriving to saturation point, from 0 to 73.8 μM. After each aliquot of GHB added, the mixture was stirred at room temperature for 20 s, and the corresponding UV–vis absorption spectrum was recorded.

For nanosensor **S2**, in a 3 mL quartz cell (1.0 cm of path length), 2800 μL of **S2** (1 mg/mL in DMSO) was mixed with increasing quantities of a 2.4 mM aqueous solution of GHB until arriving to saturation point, from 0 to 189 µM. After each aliquot of GHB added, the mixture was stirred at room temperature for 20 s, and the corresponding fluorescence emission spectra (λ_exc_ = 490 nm) were recorded.

The limits of detection (LODs) were determined using the equation: C_*LOD*_ = 3σ_b_/m (where σ_b_ is the standard deviation of the blank and m is the slope of the calibration curve). The blank signal was measured in five replicates and the standard deviation (σ_b_) was calculated. The calibration curve was obtained by preparing a series of standard solutions at different concentrations and recording their responses (the absorbance at 516 nm *vs* GHB concentration for **S1**, and the emission intensity at 534 nm *vs* GHB concentration for **S2**). The slope (m) of the calibration curve was determined through linear regression via the least squares method.

#### Selectivity studies with nanosensors S1 and S2 suspended in DMSO/H_2_O

For nanosensor **S1**, in a 3 mL quartz cell (1.0 cm of path length), 2800 μL of **S1** (0.33 mg/mL in DMSO) was mixed with 30 µL of 4.8 mM aqueous solution of the corresponding chemical submission drug used as interferent and after 20 s the corresponding UV–vis absorption spectra were recorded.

For nanosensor **S2**, in a 3 mL quartz cell (1.0 cm of path length), 2624 μL of **S2** (1 mg/mL in DMSO) was mixed with 176 µL of 3 mM aqueous solution of the corresponding chemical submission drug used as interferent, and after 20 s, the corresponding fluorescence emission spectra (λ_exc_ = 490 nm) were recorded.

#### Interference essays using nanosensors S1 and S2 in DMSO/H_2_O suspension

In a 3 mL quartz cell (1.0 cm of path length), 2800 μL of nanosensor **S1** or **S2** (0.33 mg/mL in DMSO) was mixed with 60 µL of 2.4 mM aqueous solution of the corresponding interferent. After stirring at room temperature for 20 s, the corresponding UV–vis absorption (for **S1**) or emission (for **S2**) spectra were recorded.

### Sensing experiments with S1 and S2 in solid phase

#### Solid-phase titration with nanosensor S1 with GHB in aqueous solution

The detection of GHB was visually assessed through the deposition of **S1** nanosensor on a paper previously treated with trimethylsilane groups. This silylated paper (**SP**) was prepared dipping chromatography paper fragments of approximately 10 × 2 cm in size in a 9:1 CH_2_Cl_2_:(CH_3_)_3_SiCl solution for 1 day. Then, these fragments were dried at 40 °C in an oven for 2 days more. Then, 0.5 × 0.5 cm pieces of **SP** were dipped in a solution of the nanosensor **S1** (1mg/mL in DMSO) and, after a few seconds, 15 μL of GHB aqueous solutions of different concentrations (0 to 100 mM) were dropped onto the surface of the material.

#### Solid-phase titration with nanosensor S2 with GHB in aqueous solution

The detection of GHB was visually assessed through the deposition of **S2** nanosensor on a paper previously treated with trimethylsilane groups. This silylated paper (**SP**) was prepared dipping chromatography paper fragments of approximately 10 × 2 cm in size in a 9:1 CH_2_Cl_2_:(CH_3_)_3_SiCl solution for 1 day. Then, these fragments were dried at 40 °C in an oven for 2 days more. Then, over 0.5 × 0.5 cm pieces of **SP**, 30 μL of nanosensor **S2** (1 mg/mL in H_2_O) were deposited on the **SP** and mixed with 10 μL of GHB aqueous solutions of different concentrations (0 to 50 mM).

#### Solid-phase detection of GHB with nanosensor S1 in beverages

The detection of GHB in beverages was visually assessed by dipping 0.5 × 0.5 cm pieces of **SP** in a solution of nanosensor **S1** (1 mg/mL in DMSO) and, after a few seconds, dropping 10 µL of the corresponding free-drug beverage or spiked beverage (90 mM in GHB) onto the surface of the material.

#### Solid-phase detection of GHB with nanosensor S2 in beverages

The detection of GHB in beverages was visually assessed dropping 30 μL of nanosensor **S2** solution (1 mg/mL in H_2_O) over 0.5 × 0.5 cm pieces of **SP** and, after a few seconds, dropping 10 µL of the corresponding beverage or spiked beverage (90 mM in GHB). In the case of GHB-contaminated beverages, fluorescence changes could be directly observed under a common UV-lamp.

### GHB sensing experiments in oral fluid with chemosensor 1

To test the usefulness of sensor **1**, preliminary sensing experiments were carried out on real saliva samples collected from 5 different individuals (men and women) with no observed differences. To standardize the results, the titrations were carried out on the saliva sample of one of the authors. Titrations were carried out as follows: In a 1.5 mL plastic cell (1.0 cm of path length), 500 μL of DMSO was mixed with 500 μL of the reaction mixture, previously filtered with a PTFE labfil® microfilter (pore size 0.45 μm, diam. 25 mm). The reaction mixture was prepared by mixing 1386 μL of DMSO, 94 μL of compound **1** (1 mM in DMSO), and 2 μL of HCl 0.5% with 20 μL of a GHB solution in oral fluid. All oral fluid samples were previously spiked with different quantities of GHB, obtaining solutions from 0 to 40 mM in GHB. The limit of detection (LOD) was determined using the equation: C_*LOD*_ = 3σ_b_/m (where σ_b_ is the standard deviation of the blank and m is the slope of the calibration curve). The limit of quantitation (LOQ) was calculated from the equation C_*LOQ*_ = 5σ_b_/m.

## Results and discussion

### Synthesis of chemosensor 1 and solids S1 and S2

**Scheme 1:**
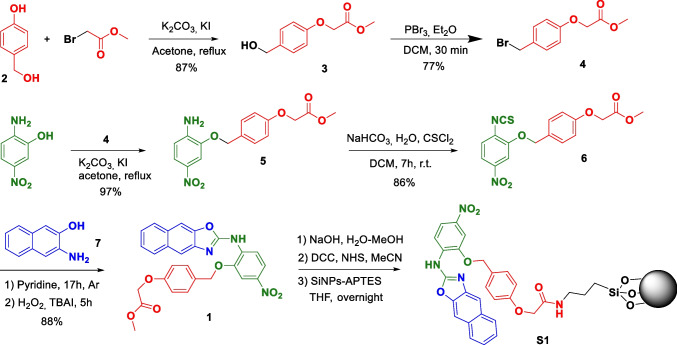
Synthetic route to obtain the chromogenic chemosensors **1** and **S1**

The synthesis of chromogenic chemosensor **1** and nanosensor **S1** was carried out as described in Scheme 1. The strategy for the design of the solid sensor **S1** was based on the use of a spacer (in red*)* which links the chromogenic unit (blue and green-colored moieties, respectively) to commercially available LUDOX.®-type silica nanoparticles (SiNPs), previously functionalized with 3-aminopropyltriethoxysilane (APTES, black-colored structure). The SiNPs-APTES nanoparticles were prepared as previously described [15].

First, 4-hydroxybenzyl alcohol (**2**) was reacted with methyl 2-bromoacetate in basic media to yield compound **3**. Then, the benzyl alcohol was transformed into the corresponding benzyl bromide by reaction with PBr_3_, yielding compound **4**. A Williamson ether synthesis between **4** and 2-amino-5-nitrophenol in basic medium led to compound **5**. The amine group in **5** was then converted to an isothiocyanate group by reaction with thionyl chloride to yield compound **6**, which was finally reacted with 3-amino-2-naphthol (**7**) to yield compound **1**, incorporating a naphthoxazole unit connected to a *p*-nitrophenyl derivative, and a labile methyl ester. The ester group in **1** was then hydrolyzed to the corresponding carboxylic acid, which was made to react with the previously prepared SiNPs-APTES, in the presence of N,N’-dicyclohexylcarbodiimide (DCC) and N-hydroxysuccinimide (NHS) as coupling agents, to yield solid **S1**. All intermediates were confirmed and characterized by ^1^H and ^13^C-NMR, FTIR and HRMS (see Supplementary Information).

**Scheme 2:**
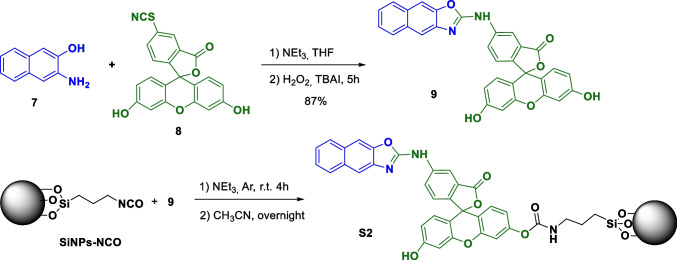
Synthesis of nanosensor **S2**

The synthesis of the fluorogenic nanosensor **S2** was carried out following the synthetic pathway described in Scheme 2. First, chromogenic unit **9** was prepared according to the procedure described in the literature, from 3-amino-2-naphthol (**7**) and fluorescein isothiocyanate **8** to obtain a naphthoxazole unit in a single step, in 87% yield [12]. On the other hand, isocyanate-ended LUDOX®-type silica nanoparticles (SiNPs-NCO) were prepared as previously reported [16]. Then, SiNPs-NCO nanoparticles were reacted with compound **9** through one of its phenolic groups to yield **S2** in which the chromogenic unit is covalently attached to the silica nanoparticles through a carbamate bond.

**Fig. 1:**
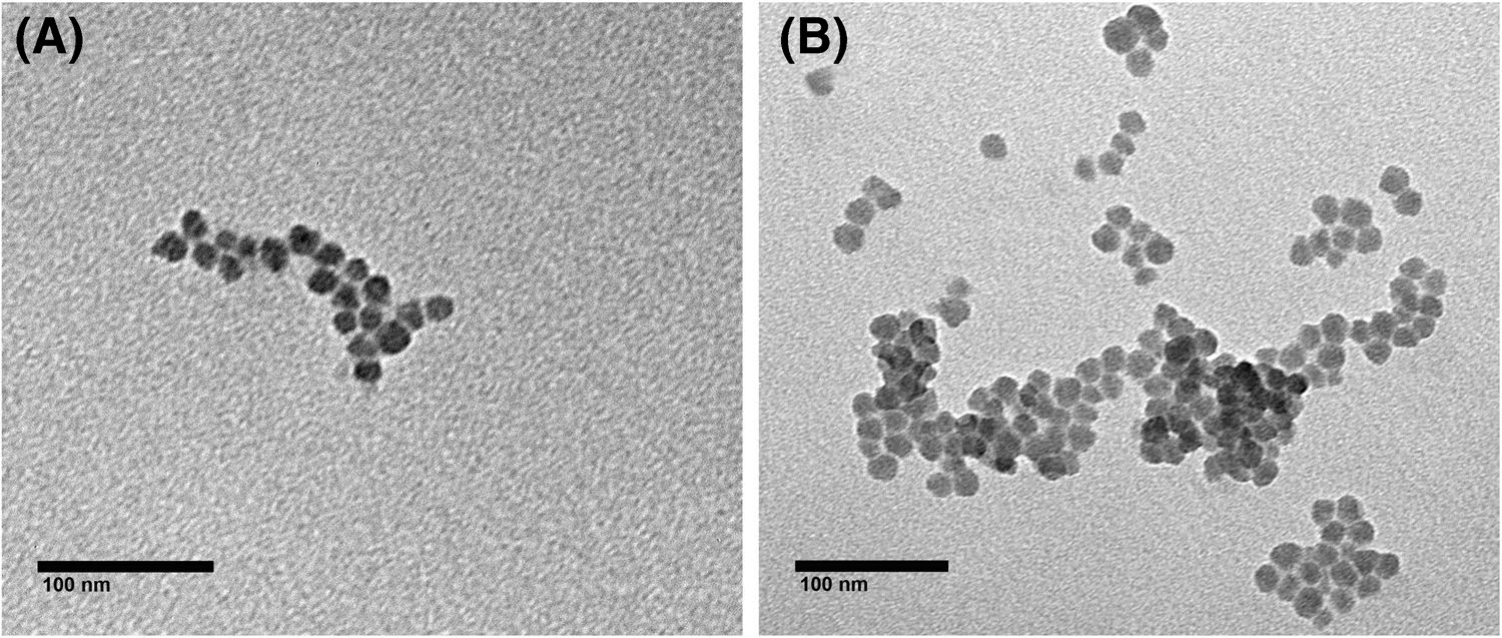
TEM images of solids (**A**) **S1** and (**B**) **S2**

Solids SiNPs-APTES, SiNPs-NCO, **S1** and **S2** were characterized by standard techniques, such as thermogravimetric analysis (TGA), dynamic light scattering (DLS) or transmission electron microscopy (TEM). TGA shows 0.24 mmol of chemosensor per gram of solid **S1** and 0.14 mmol/g of solid **S2** (Fig. S14). The zeta potential values are −36.6 ± 9.7 and 28.8 ± 5.4 mV for **S1** and the SiNPs-APTES nanoparticles respectively, whereas the zeta potential values obtained for SiNPs-NCO and **S2** were, 27.6 ± 5.2, and −28.8 ± 8.6 mV respectively (Fig. S15), confirming that the coupling reactions took place correctly. In addition, hydrodynamic diameter values of 100 ± 0.6 nm for **S1** and 115.8 ± 1 nm for **S2** were found through dynamic light scattering (DLS) analysis (Fig. S16). Finally, TEM images for **S1** and **S2** (Fig. 1) showed good dispersity and a very homogeneous particle size (16.2 ± 1.9 nm for **S1** and 16.6 ± 2.0 nm for **S2**).

### Sensing experiments in solution with suspensions of the nanosensors

Prior to testing the two nanosensors **S1** and **S2** in complex matrices, such as beverages and saliva, an evaluation of the spectroscopic properties of these devices and their ability to detect GHB in aqueous dimethyl sulfoxide (DMSO) solution, using the solids as nanoparticles suspensions, was performed.

**Fig. 2:**
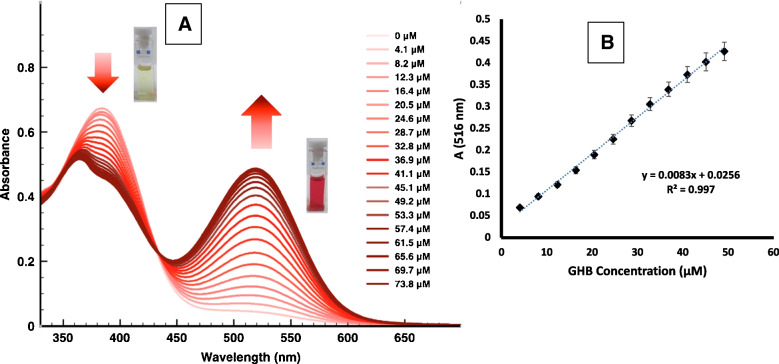
**A** UV–vis absorption spectra of nanosensor **S1** (0.33 mg/ml in DMSO) with increasing amounts of GHB (0 to 73.8 μM). **B** Linear calibration curve representing the absorption intensity at λ = 516 nm *vs* the concentration of GHB

The UV–vis absorption spectrum of chromogenic nanosensor of **S1** in DMSO shows a maximum absorption band at 370 nm and a small shoulder at 516 nm, yielding pale yellow solutions. On the other hand, fluorogenic nanosensor **S2** in DMSO is almost non-fluorescent, with a weak emission band at 534 nm (λ_exc_ = 490 nm). In the presence of GHB, the UV–vis spectrum of **S1** shows a decrease in the intensity of the band at 370 nm and the appearance of a new absorption band at 516 nm, which increases in the presence of increasing amounts of GHB, resulting in colour shift of the solution from pale yellow to red clearly visible to the naked eye (Fig. 2). On the other hand, in the presence of GHB, **S2** solutions show an important increase of the 534 nm emission band. These spectra both in the absence and presence of GHB were stable over time at the indicated concentrations. Regarding the sensing mechanism, we believe that GHB-induced deprotonation of the 2-aminonaphthoxazole-based probes, as previously reported [12], is responsible for the observed changes in their chromo-fluorogenic response.

Titration of a suspension of **S1** in DMSO (0.33 mg/mL) with increasing amounts of an aqueous GHB solution was monitored by UV–vis absorption spectroscopy (see Fig. 2). The absorbance band at 370 nm decreased while the new absorbance band at 516 nm increased with the addition of GHB (Fig. 2A), registering an isosbestic point at 440 nm. In this case, the absorbance at 516 nm varies linearly with GHB concentration (Fig. 2B) with R.^2^ = 0.997. A limit of detection (LOD) of 2.21 μM of GHB was determined using the equation C_*LOD*_ = 3σ_b_/m (where σ_b_ is the standard deviation of the blank and m is the slope of the calibration curve). It is noteworthy to remark that this is a value substantially lower than the concentration of GHB in spiked beverages necessary to cause harmful/sedative effects, which is around 96 mM [17].

**Fig. 3:**
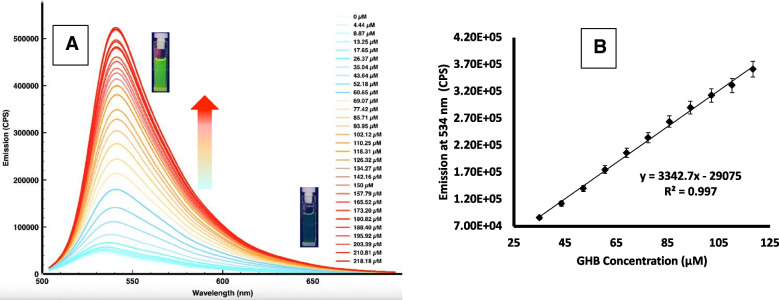
**A** Emission spectra of nanosensor **S2** (1 mg/ml in DMSO, λ_exc_ = 490 nm) with increasing amounts of GHB (from 0 to 218.2 μM). **B** Calibration curve representing the emission intensity at λ = 534 nm *vs* the concentration of GHB

In a similar way, titration of **S2** in DMSO with increasing amounts of aqueous GHB solution was monitored by fluorescence emission spectroscopy. The emission band at 534 nm (λ_exc_ = 490 nm) increases linearly with the concentration of GHB (Fig. 3A and B) The LOD calculated for this nanosensor was 1.65 μM of GHB. Our nanosensors compare satisfactorily with recently reported optical probes for GHB, especially in terms of LOD and response time, as shown in Table S3.

**Fig. 4:**
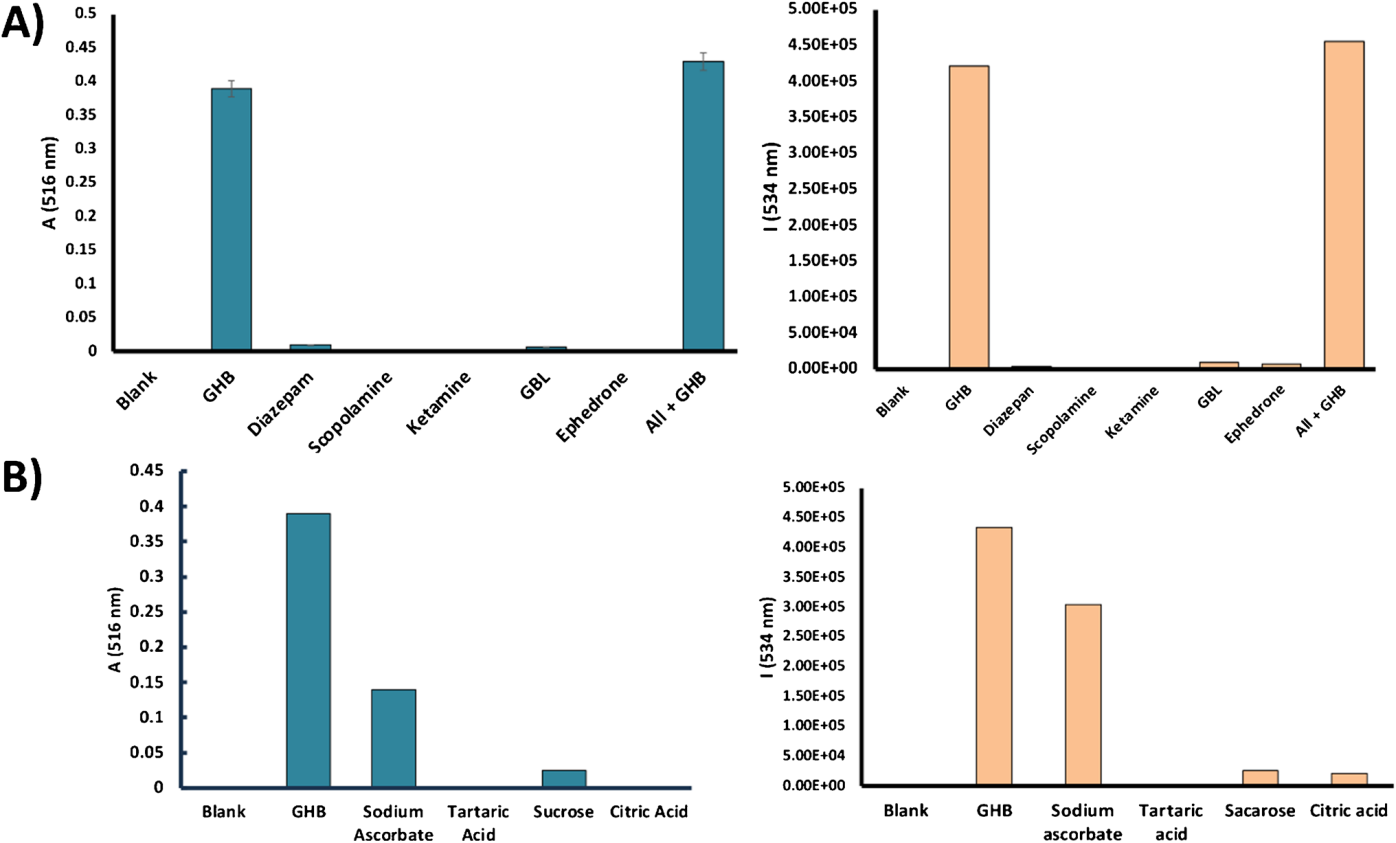
**A** (Left) Absorption at λ = 516 nm of nanosensor **S1** (0.33 mg/mL in DMSO) samples spiked with different chemical submission drugs (49.2 μM). (Right) Emission intensity at λ = 534 nm (λ_exc_ = 490 nm) of nanosensor **S2** (1 mg/mL in DMSO) samples spiked with different chemical submission drugs (189 μM). **B** (Left) Absorption at λ = 516 nm of nanosensor **S1** (0.33 mg/mL in DMSO) and (right) emission intensity at λ = 534 nm (λ_exc_ = 490 nm) of nanosensor **S2** (1 mg/mL in DMSO) solutions containing GHB or different compounds usually present in soft drinks (49.2 μM for **S1** and 189 μM for **S2**)

After the spectroscopic characterization of the **S1** and **S2** nanosensors and testing their sensitivity in the detection of GHB, their selectivity was tested using other substances employed for chemical submission, such as scopolamine, ketamine, diazepam and ephedrone. Figure 4 reveals that no discernible changes in the absorbance (**S1**, Fig. 4, left) and emission (**S2**, Fig. 4, right) properties of the corresponding nanosensors, were observed in the presence of the previously mentioned drugs. Furthermore, the absorbance (nanosensor **S1**) or fluorescence (nanosensor **S2**) observed intensities, in a sample containing all interferents at the same time, was similar to that recorded only in the presence of GHB (see Fig. 4A). It is important to remark that the concentration of these substances in all samples was higher than those required to induce chemical submission (96 mM) [17].

Having explored the selectivity of **S1** and **S2** nanosensors against GHB in simple matrices, the possibility of using these nanosensors in different soft drinks spiked with this drug was studied. For this purpose, the interfering potential of several compounds present in these beverages, such as acids (tartaric and citric acids), reducing species (sodium ascorbate) and sugars (sucrose) was tested. Figure 4B shows the response of **S1** and **S2** solutions against GHB and different interferents at the same concentration. Only sodium ascorbate interferes in the absorption (**S1**) or emission (**S2**) response of these nanosensors, but its signal is lower than that observed for GHB. Finally, other possible interferents with a structure similar to that of GHB, such as butanediol, 1, 4-butanediol diacetate and GABA were also tested (see Figs. S17 and S18). Of these, only GABA gave some response to probe **S1**, although much lower than GHB.

### Sensing experiments in solid phase

Since our goal was to obtain a portable and fast-responding solid phase device, we decided to deposit **S1** and **S2** nanosensors on different solid supports. The first thing we tried was to deposit them on regular cellulosic paper dipping fragments of it into THF solutions of the corresponding nanosensor, since this support had already given us good results [18]. Although a moderate response from both **S1** and **S2** systems to the presence of GHB was obtained, the results were improved if the paper was previously hydrophobized through a silylation process. To obtain this silylated paper (**SP**), 10 × 2 cm fragments of chromatography paper were immersed in a 9:1 CH_2_Cl_2_:(CH_3_)_3_SiCl solution for 1 day. Once dried, the **S1** and **S2** sensors could be deposited over **SP**, by a dipping process, to test the presence of GHB on different samples (see Experimental Section).

**Fig. 5:**
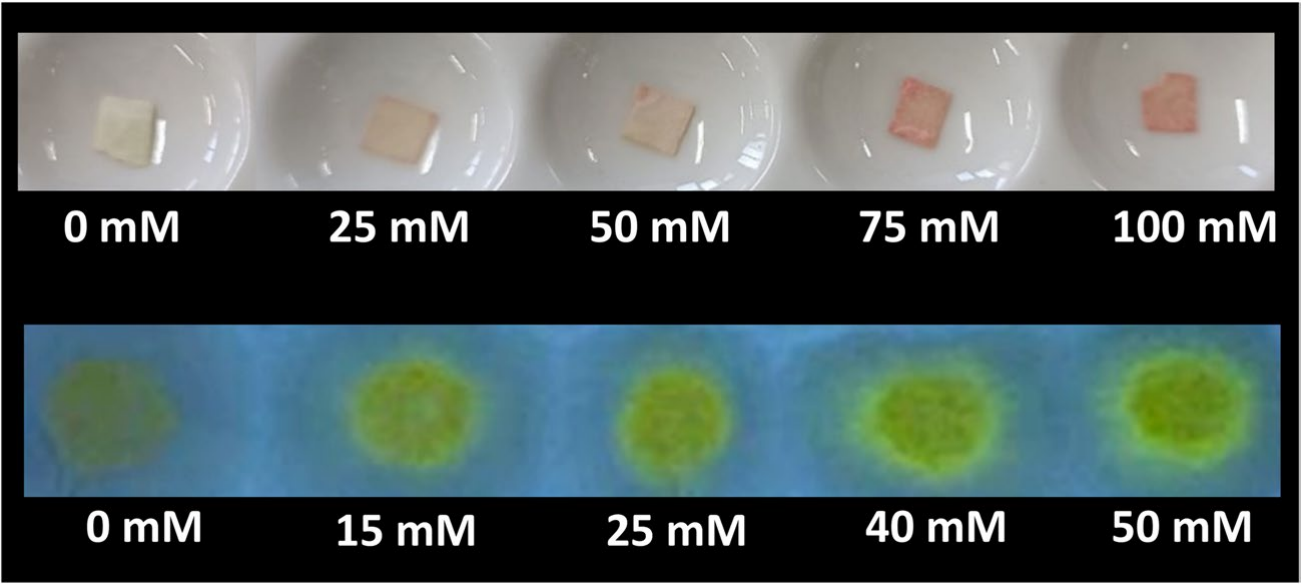
(top row) Color changes observed by the naked eye on pieces of **SP** previously dipped in a solution of nanosensor **S1** (1 mg/mL in DMSO), with 15 µL of GHB aqueous solution of different concentrations. (bottom row) Fluorescence changes observed by the naked eye under a common UV light lamp on pieces of **SP** where 30 µL of nanosensor **S2** (1 mg/mL in H_2_O) have been deposited and 10 µL of GHB aqueous solutions of different concentrations have been added

As can be seen in Fig. 5, the color or fluorescence changes in **SP** can be directly observed by the naked eye under daylight (**S1**) or under a common UV-lamp (**S2**) at GHB concentrations above 25 and 15 mM respectively.

**Fig. 6:**
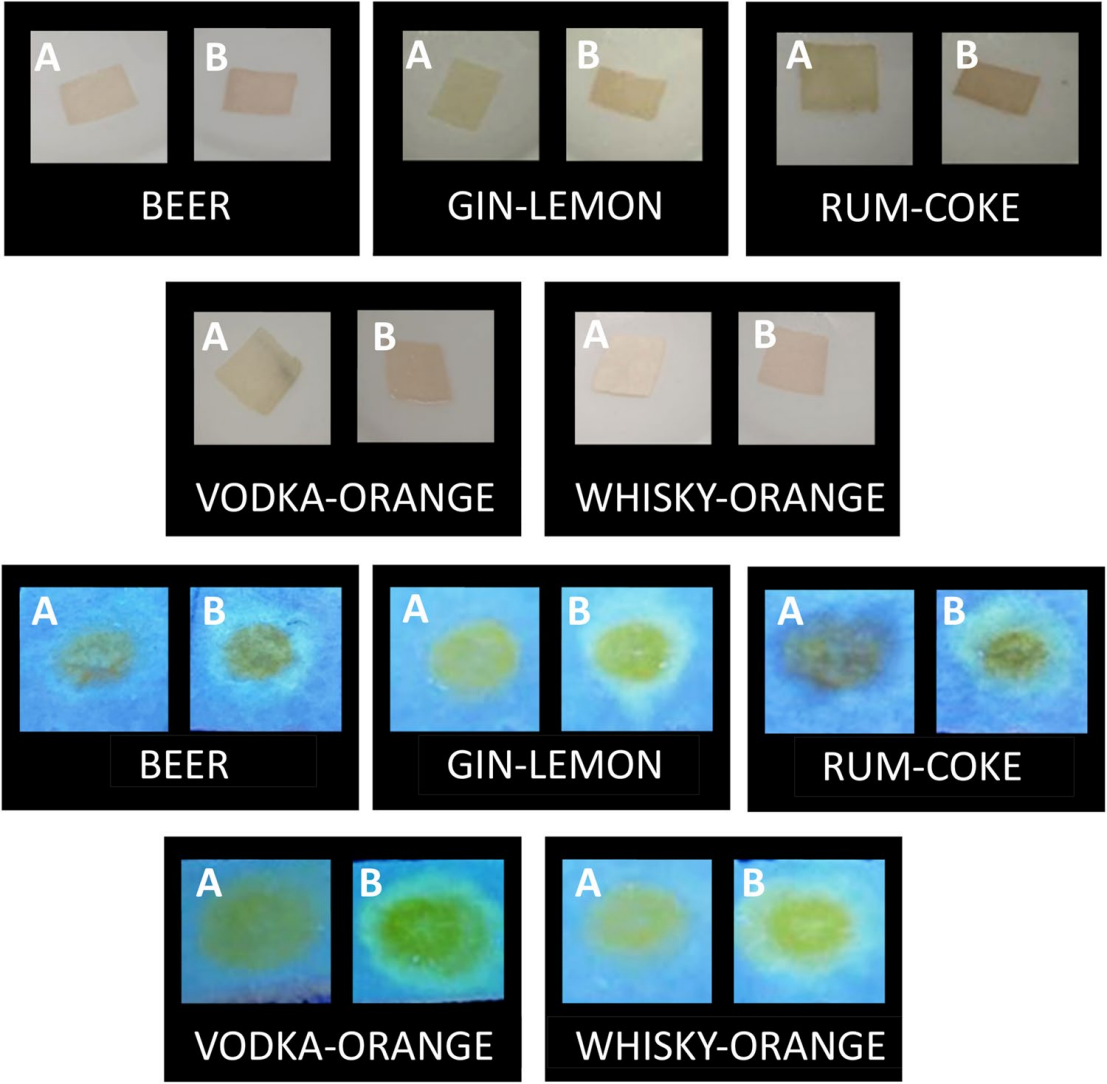
(Up) Color changes observed by the naked eye on **SP** fragments previously dipped in a solution of nanosensor **S1** (1 mg/mL in DMSO) where 15 µL of non-spiked (**A**) and spiked (**B**) beverages were deposited. (Down) Fluorescence changes observed under UV light lamp on **SP** fragments where 40 µL of nanosensor **S2** solution (1 mg/mL in H_2_O) were deposited and 15 µL of non-spiked (**A**) and spiked (**B**) beverages were added

The results obtained prompted us to test the chromogenic or fluorescent response of this **SP** with either **S1** or **S2** nanosensor towards alcoholic and non-alcoholic beverages spiked with GHB (see Experimental Section for details). The color (**S1,** Fig. 6, up) and fluorescence (**S2**, Fig. 6, down) changes observed on **SP** upon contact with GHB-spiked samples were immediate.

### Sensing experiments in saliva with chemosensor 1

**Fig. 7:**
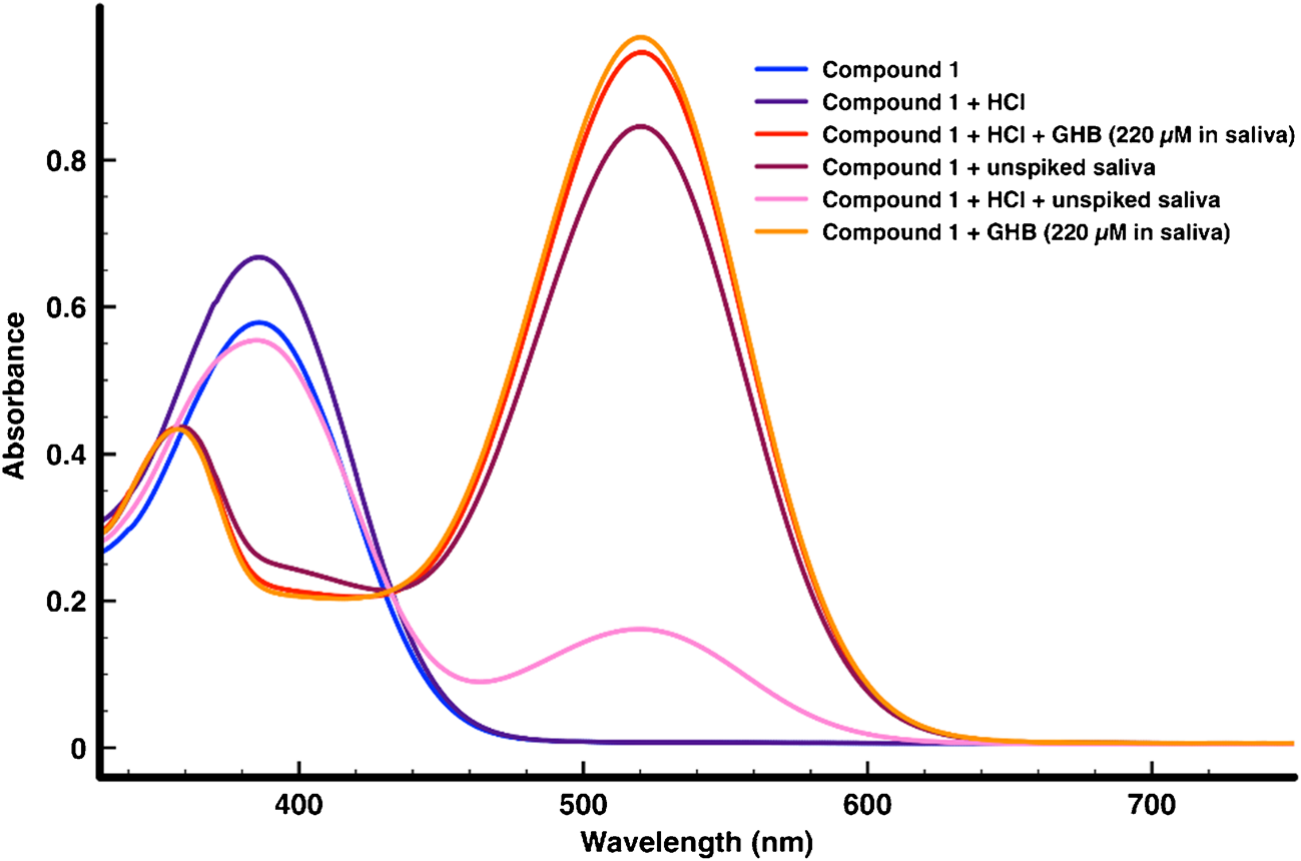
UV–vis absorption spectra of compound **1** (31.25 μM in DMSO); compound **1** (31.25 μM in DMSO) + 2 μL of aqueous HCl 0.5%; compound **1** (31.25 μM in DMSO) in unspiked saliva; compound **1** (31.25 μM in DMSO) + 2μL of aqueous HCl 0.5% in unspiked saliva; and compound **1** (31.25 μM in DMSO) + 2μL of aqueous HCl 0.5% in GHB-spiked saliva; compound **1** (31.25 μM in DMSO) in GHB-spiked saliva

Having tested the response of **S1** and **S2** nanosensors on **SP** towards GHB in alcoholic beverages, we decided to explore the detection of GHB in a complex biological matrix such as saliva, to enable the possibility of its usage in forensic studies or to pre-triage potential victims of GHB-mediated chemical submission. The possibility of detecting this drug in oral fluid in a rapid and easy way would be of great importance for forensic studies [19]. For this, we tested compound **1** as a chromogenic chemosensor for GHB. In our preliminary experiments with saliva, we were discouraged to observe that the UV–vis spectrum of compound **1** (in the absence of GHB) diverges from that in DMSO, showing a strong absorption at 516 nm (see Fig. 7). Since the response of the sensor is caused by deprotonation, we suspected that the basicity of the oral fluid could be responsible for this effect. So, some experiments were conducted by adding small amounts of HCl to the saliva prior to adding GHB. After optimizing the measuring conditions, we were delighted to observe that in slight acidic media the sensor **1** gave a very little response in saliva, in the absence of GHB. However, in the presence of GHB, an intense absorption at 516 nm could be observed, with the corresponding change in the color of the solution, from very pale pinkish to a strong pink color. The best results were obtained with a 31.25 μM solution of compound **1** in DMSO in the presence of 2 μL of aqueous HCl 0.5% and GHB-spiked saliva, previously filtered with a microfilter (see Experimental Section). These conditions allowed a clear visualization of the color change due to GHB contamination in saliva as observed in Fig. 7.

**Fig. 8:**
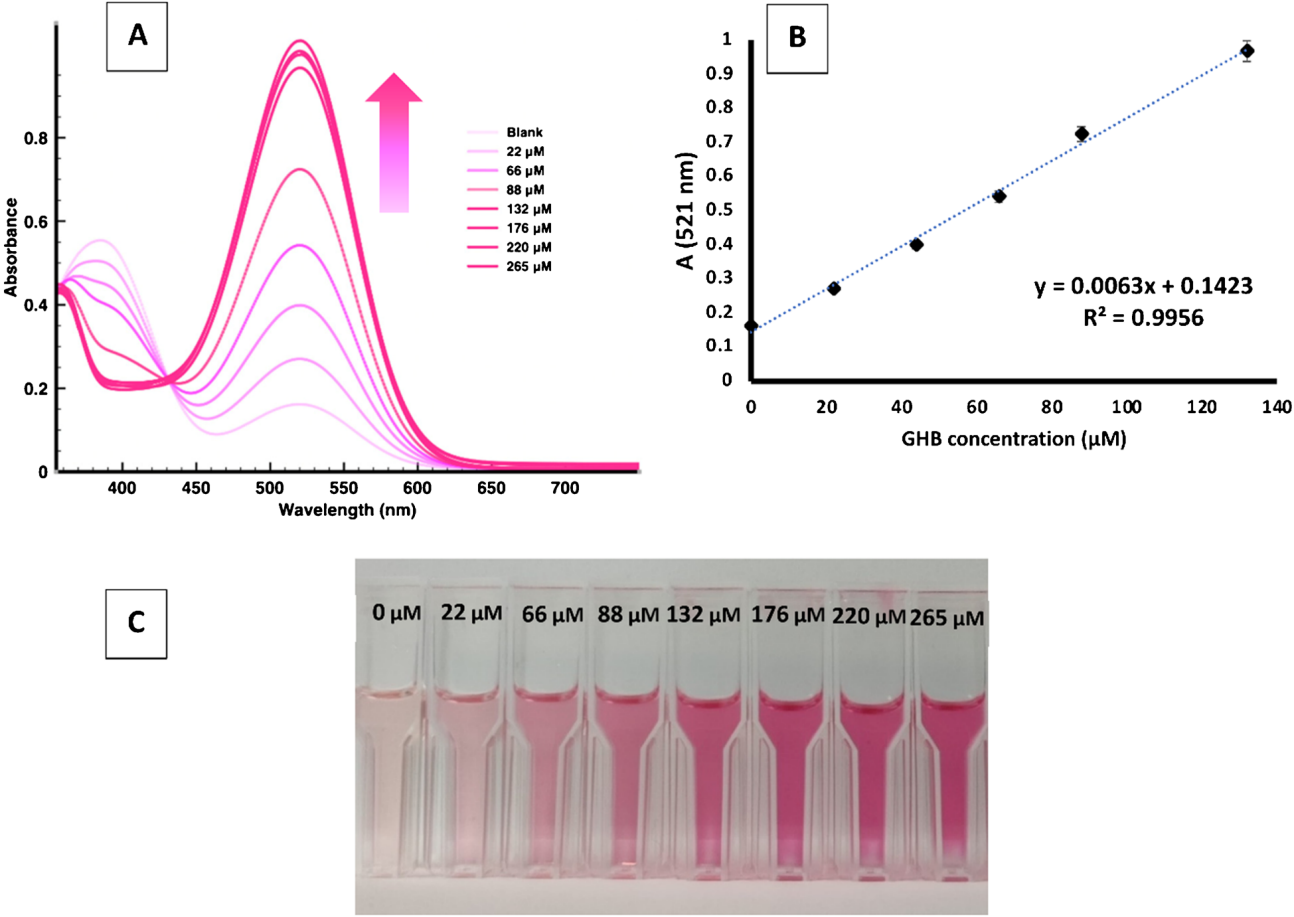
**A** Absorbance spectra of compound **1** (31.25 μM in DMSO) + 2 μL of aqueous HCl 0.5%, and increasing amounts of GHB, from 0 to 265 μM. **B** Calibration curve representing absorption intensity at λ = 521 nm *vs* the concentration of GHB. **C** Color changes observed for compound **1** under the optimized conditions with increasing amounts of GHB

Using these optimized conditions, the titration of compound **1** in DMSO with increasing amounts of GHB in aqueous solution was performed and monitored by UV–vis spectroscopy. As the GHB concentration increases, the absorbance of the band at 370 nm decreases and that of the band at 516 nm increases (see Fig. 8A). The absorbance at 516 nm varies linearly with GHB concentration in the 32–132 μM range, with R^2^ = 0.9970 (Fig. 8B). From the linear calibration curve, a LOQ of 32 μM and a LOD 19.2 μM of GHB could be determined (see Experimental section). The calculated LOD falls within the cutoff levels of GHB for saliva (reported endogenous GHB levels in saliva are in the range 4–30 μM) [20] and is substantially lower than that expected in the saliva of victims of chemical submission due to GHB ingestion.

**Fig. 9:**
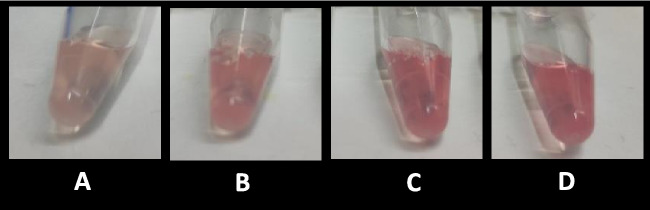
Color changes observed by unaided eye: compound **1** (62.5 μM in DMSO) + 2 μL of HCl 0.5% in aqueous solution, and 20 μL of saliva aqueous solution spiked with increasing GHB amounts: 0 mM (**A**); 0.4 mM (**B**); 0.8 mM (**C**) and 1.6 mM (**D**)

Once these studies with chemosensor **1** in saliva were completed, rapid tests were carried out in saliva taken directly from the mouth under the same conditions indicated above, but without the filtering and dilution steps, resulting in a rapid and reliable color change directly observed by the naked eye (Fig. 9).

## Conclusions

In conclusion, two new two silica-based optical chemosensors, **S1** and **S2**, for rapid, selective and on-site detection of the chemical submission drug γ-hydroxybutyrate (GHB) in beverages, have been synthesized and fully characterized. Both nanosensors consist of silica nanoparticles incorporating on their surface a 2-aminonaphthoxazole unit connected to either a *p*-nitrophenyl (**S1**) or a fluorescein (**S2**) moiety for the chromogenic or fluorogenic detection of GHB respectively. Nanosensors **S1** and **S2** demonstrated exceptional sensitivity, specificity, and versatility, enabling reliable optical detection of GHB, with LODs of 2.21 and 1.65 μM for **S1** and **S2** respectively. These LODs are much lower than the usual amount of drug in beverages necessary to induce chemical submission. Changes in solution were observed immediately by the naked eye, with minimal interferences from other substances typically found in beverages.

To improve the portability of these sensors, they could be integrated into portable solid-phase devices through their deposition on silylated cellulose paper. This allowed for immediate solid-phase detection of the presence of GHB in real beverages through a change in the color of the sensor or the appearance of yellow fluorescence under a common UV-light, enhancing their practicality for real-world applications, such as nightlife venues or forensic settings.

Finally, a 2-aminonaphthoxazole-based chemosensor **1** for the visual detection of GHB in saliva has been synthesized and evaluated. Chemosensor **1** was able to immediately detect the presence of GHB in oral fluid by the appearance of a pink colour in the solution that could be observed by the naked eye. The UV–vis response of **1** towards GHB showed a high linearity in the 32–132 μM range, with a calculated LOD of 19.2 μM. This LOD falls within the endogenous GHB levels in saliva and therefore, a higher concentration of GHB in saliva is indicative of consumption of these drugs.

We believe that our work represents a transformative step in drug sensing, bridging a critical gap in GHB detection technology, providing accessible tools for GHB detection in beverages and oral fluid and supporting efforts to combat drug-facilitated crimes.

## Supplementary Information

Below is the link to the electronic supplementary material.

Supplementary file1 (PDF 1483 KB)

## Data Availability

No datasets were generated or analysed during the current study.
