# Supplementary material for: Silica-based optical chemosensors for rapid and reliable on-site detection of gamma-hydroxybutyrate in beverages and oral fluids
Source: Mikrochim Acta. 2025 Apr 2;192(5):275. doi: 10.1007/s00604-025-07134-9 (PMC11965150; doi:10.1007/s00604-025-07134-9)
Supplement: Supplementary file 1 — Supplementary file1 (PDF 1483 KB) [file 604_2025_7134_MOESM1_ESM.pdf]

Silica-Based Optical Chemosensors for Rapid and Reliable On-Site Detection of GHB in Beverages and Oral Fluids

Jordi Hernández-Contreras[1], Jordi Roig-Rubio[1], Margarita Parra[1,2,3], Salvador Gil[1,2,3], Pau Arroyo[1,2], José A. Sáez[1,2], Carlos Lodeiro[4,5], Pablo Gaviña[1,2,3,*]

[1] Instituto Interuniversitario de Investigación de Reconocimiento Molecular y Desarrollo Tecnológico (IDM). Universitat Politècnica de València, Universitat de València, Doctor Moliner 50, Burjassot, 46100, Valencia.
[2] Departamento de Química Orgánica, Universitat de València, Doctor Moliner 50, Burjassot, 46100, Valencia, Spain.
[3] CIBER de Bioingeniería, Biomateriales y Nanomedicina (CIBER-BBN), Spain.
[4] BIOSCOPE Research Group, LAQV-REQUIMTE, Chemistry Department, NOVA School of Science and Technology, NOVA FCT, Universidade NOVA de Lisboa, 2829-516 Caparica, Portugal.
[5] PROTEOMASS Scientific Society, Costa de Caparica, 2825-466, Portugal

Supplementary Information

Contents

Figure S1. *^{1}H NMR spectrum of compound **3** in DMSO-d_6*........ 3

Figure S2. *^{1}H NMR spectrum of compound **4** in $CDCl_3$*........ 3

Figure S3. *^{13}C NMR spectrum of compound **4** in $CDCl_3$*........ 4

Figure S4. *^{1}H NMR spectrum of compound **5** in $CDCl_3$*........ 4

Figure S5. *^{13}C NMR spectrum of compound **5** in $CDCl_3$*........ 5

Figure S6. *^{1}H NMR spectrum of compound **6** in $CDCl_3$*........ 5

Figure S7. *^{13}C NMR spectrum of compound **6** in $CDCl_3$*........ 6

Figure S8. *^{1}H NMR spectrum of compound **1** in $CDCl_3$*........ 6

Figure S9. *^{13}C NMR spectrum of compound **1** in $CDCl_3$*........ 7

Figure S10. *Mass spectroscopy data of compound **1***........ 7

Figure S11. *^{1}H NMR spectrum of compound **9** in DMSO-d_6*........ 8

Figure S12. *^{13}C NMR spectrum of compound **9** in DMSO-d_6*........ 8

Figure S13. *Mass spectroscopy data of compound **9***........ 9

Figure S14. *Thermogravimetric analysis (TGA) of solids **S1** and **S2***........ 9

Figure S15. *Zeta Potential for **SiNPs-APTES** (depicted as "NP-NH2"), **S1**, **SiNPs-NCO** (depicted as "NP-NCO") and **S2***... 10

Figure S16. *Hydrodynamic diameter distribution of **S1** (green line) and **S2** (orange line)*........ 10

Figure S17. *Absorption at λ = 516 nm of nanosensor **S1** (0.33 mg/mL in DMSO) solutions containing GHB and different possible interferents (49.2 µM)*........ 11

Figure S18. *Emission intensity at λ = 534 nm (λ_{exc} = 490 nm) of nanosensor **S2** (1 mg/mL in DMSO) solutions containing GHB and different possible interferents (189 µM)*........ 11

Table S1. *Recovery and accuracy of the method with **S1** to detect GHB in aqueous samples were calculated according to reference K. A. Rawat, R. K. Singhal, S. K. Kailasa, RSC Adv. 6 (2016) 32025-32036. https://doi.org/10.1039/c6ra01575a* *12*

Table S2. *Recovery and accuracy of the method with **S2** to detect GHB in aqueous samples were calculated according to reference K. A. Rawat, R. K. Singhal, S. K. Kailasa, RSC Adv. 6 (2016) 32025-32036. https://doi.org/10.1039/c6ra01575a* *12*

Table S3. *Comparison of the sensing properties of different optical selective probes for GHB detection*............................ *12*

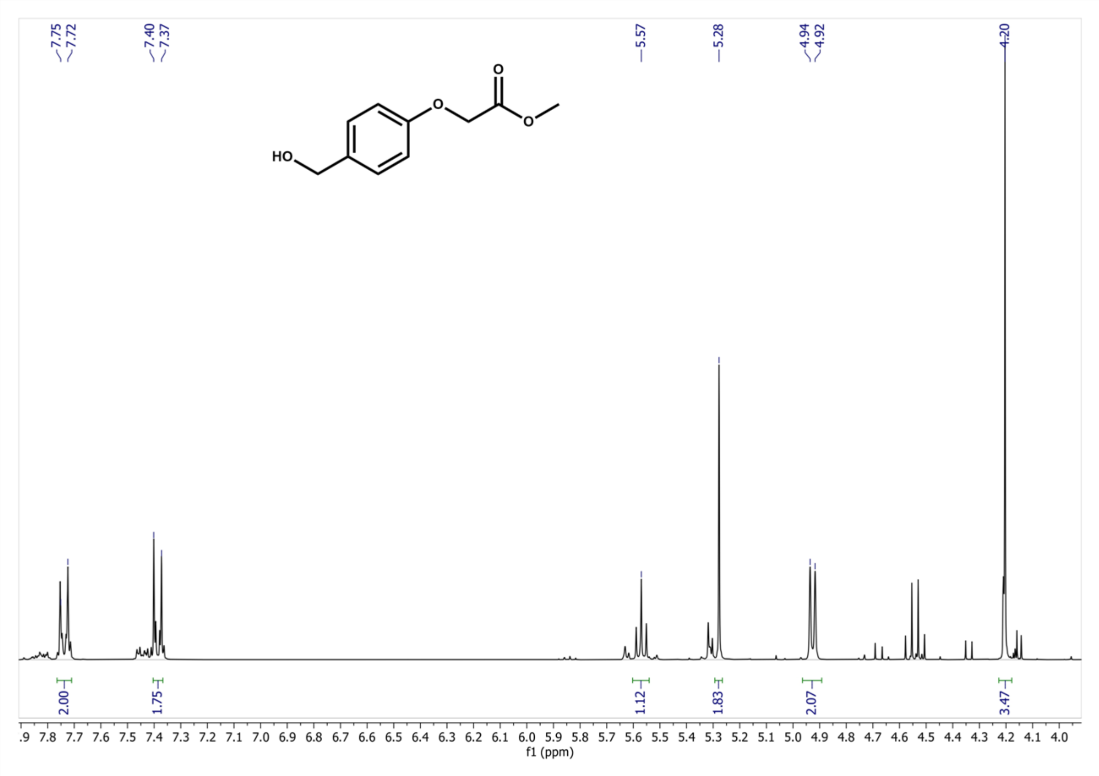

Figure S1. ^{1}H NMR spectrum of compound **3** in DMSO-d_6.

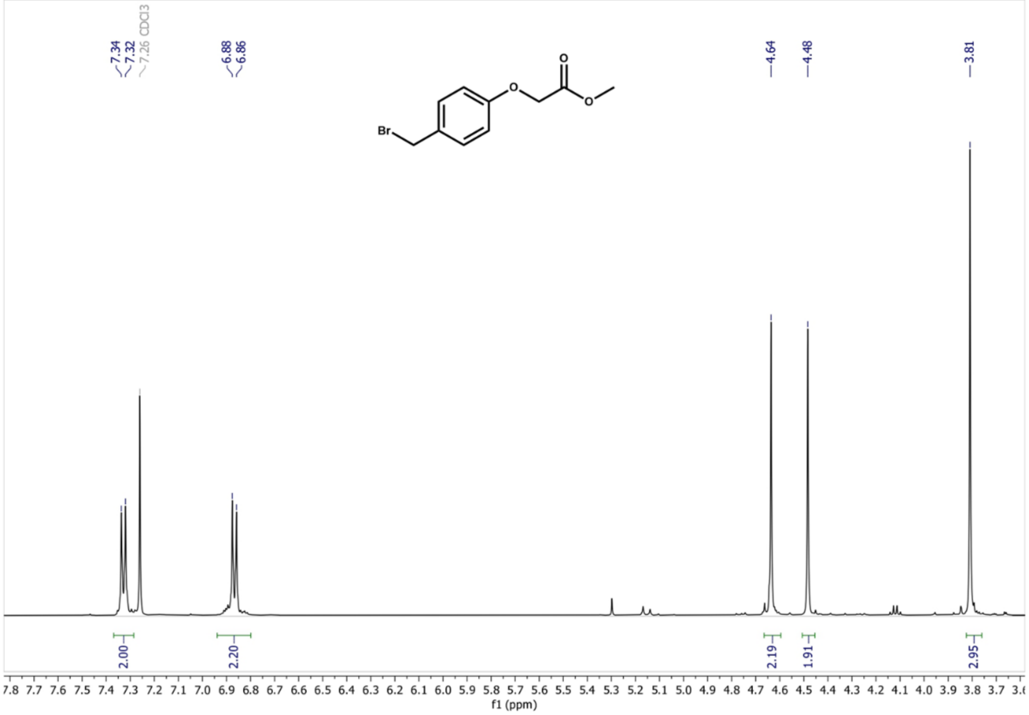

Figure S2. ^{1}H NMR spectrum of compound **4** in $CDCl_3$.

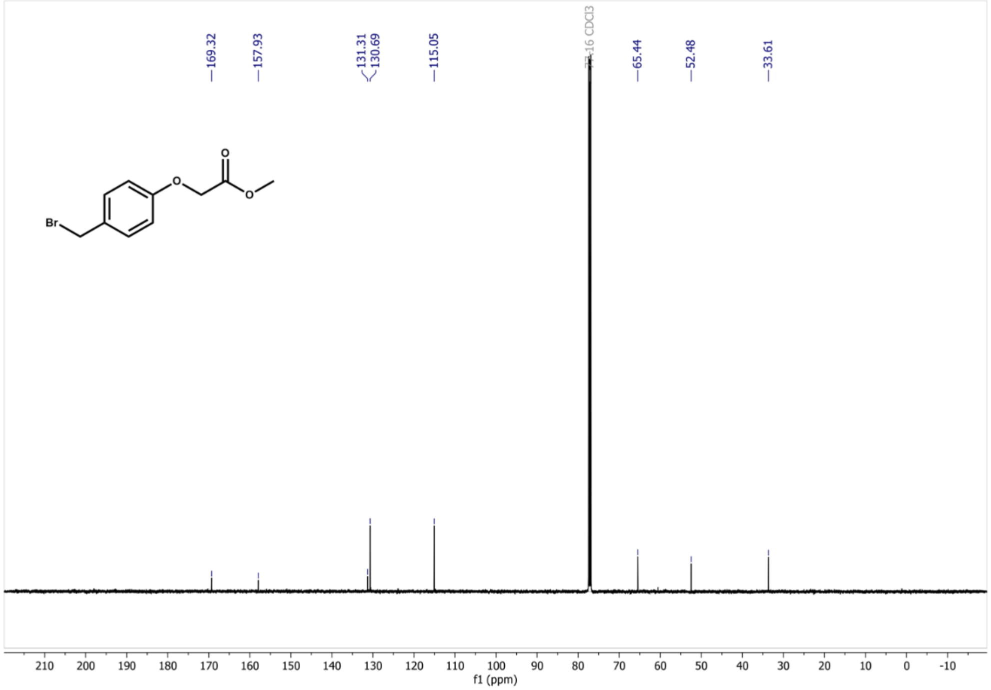

Figure S3. ^{13}C NMR spectrum of compound **4** in $CDCl_3$.

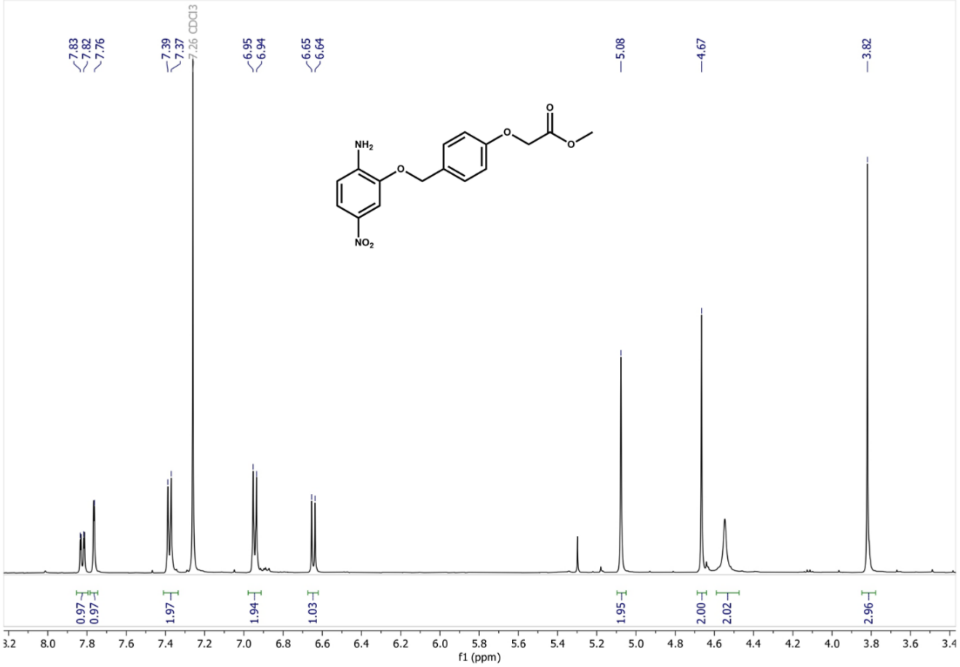

Figure S4. ^{1}H NMR spectrum of compound **5** in $CDCl_3$.

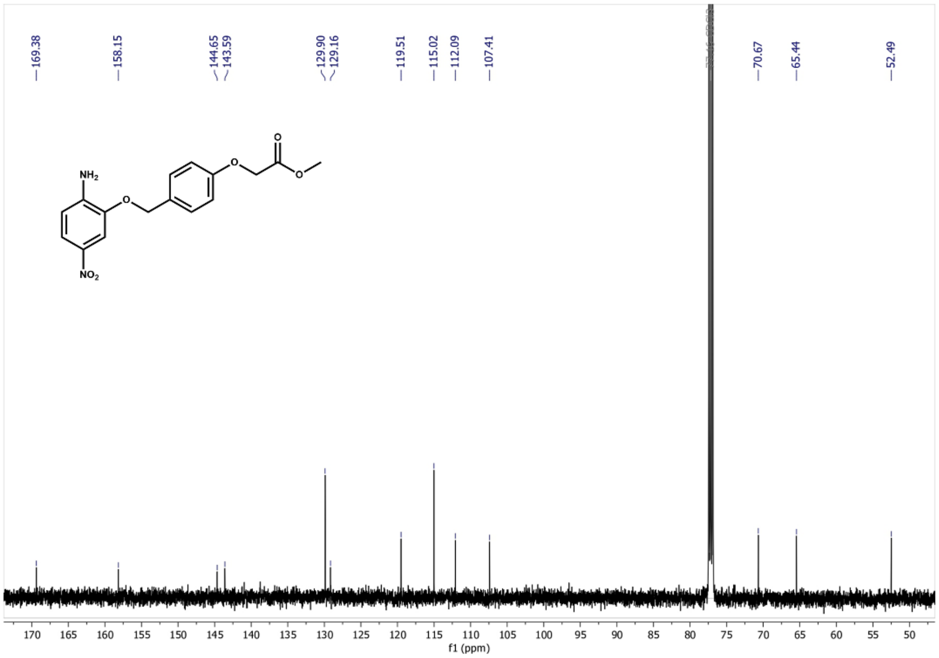

Figure S5. ^{13}C NMR spectrum of compound **5** in $CDCl_3$.

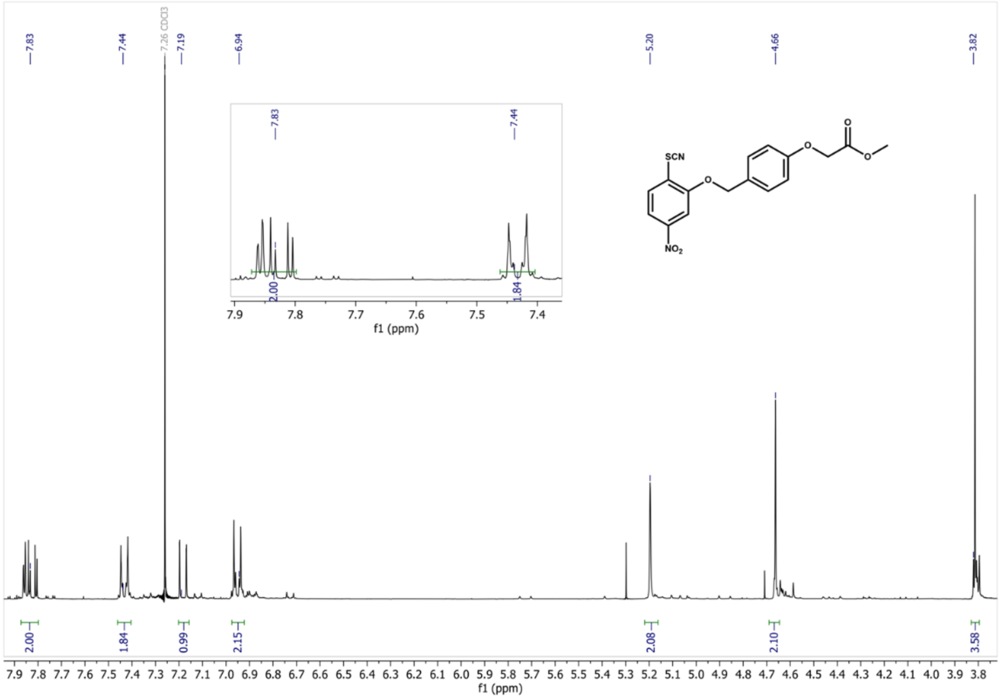

Figure S6. ^{1}H NMR spectrum of compound **6** in $CDCl_3$.

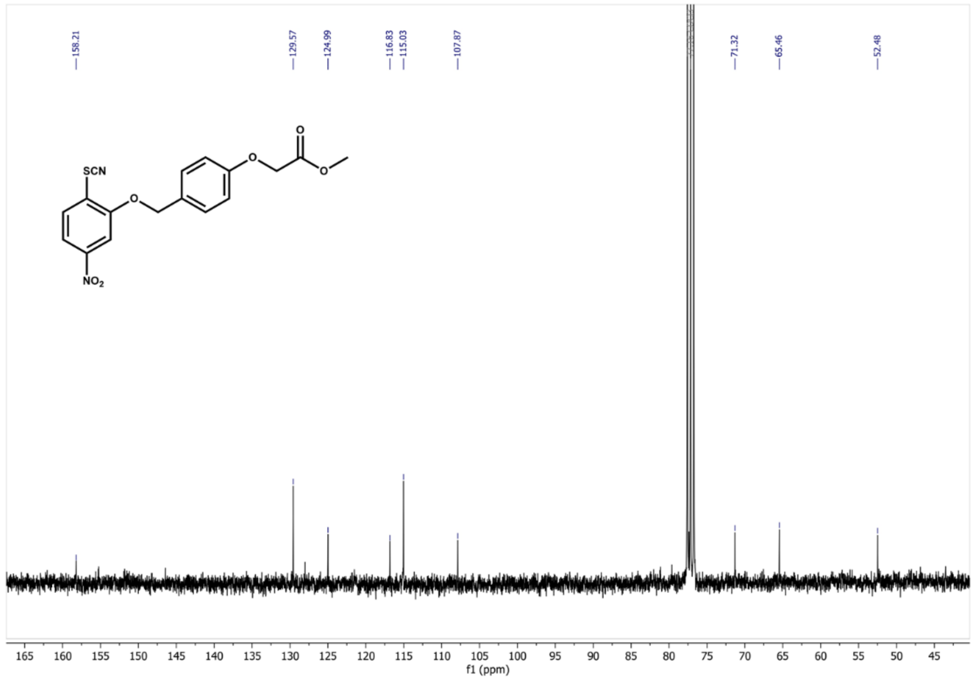

Figure S7. ^{13}C NMR spectrum of compound **6** in $CDCl_3$.

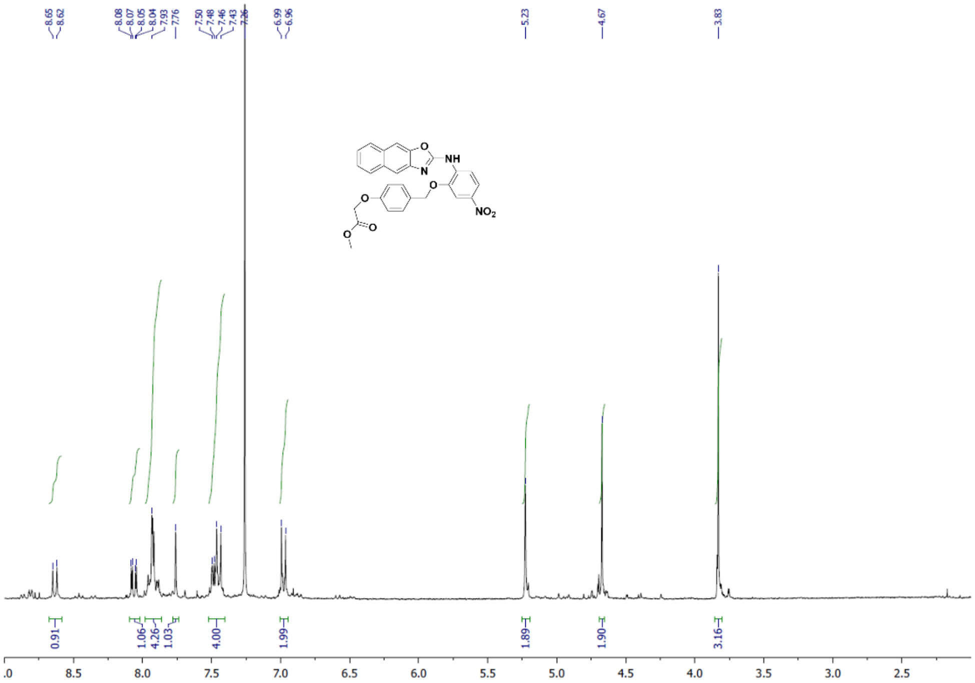

Figure S8. ^{1}H NMR spectrum of compound **1** in $CDCl_3$.

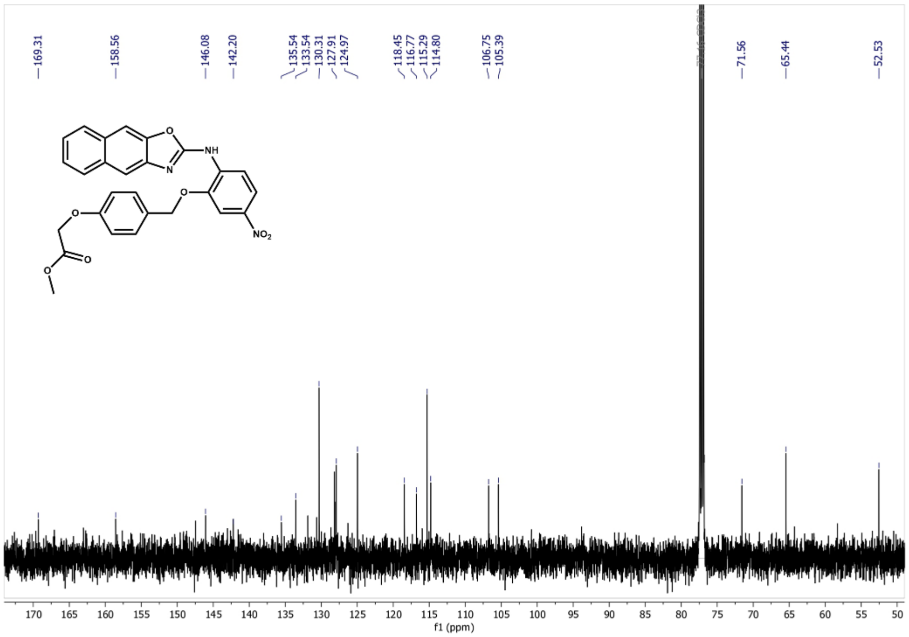

Figure S9. ^{13}C NMR spectrum of compound **1** in $CDCl_3$.

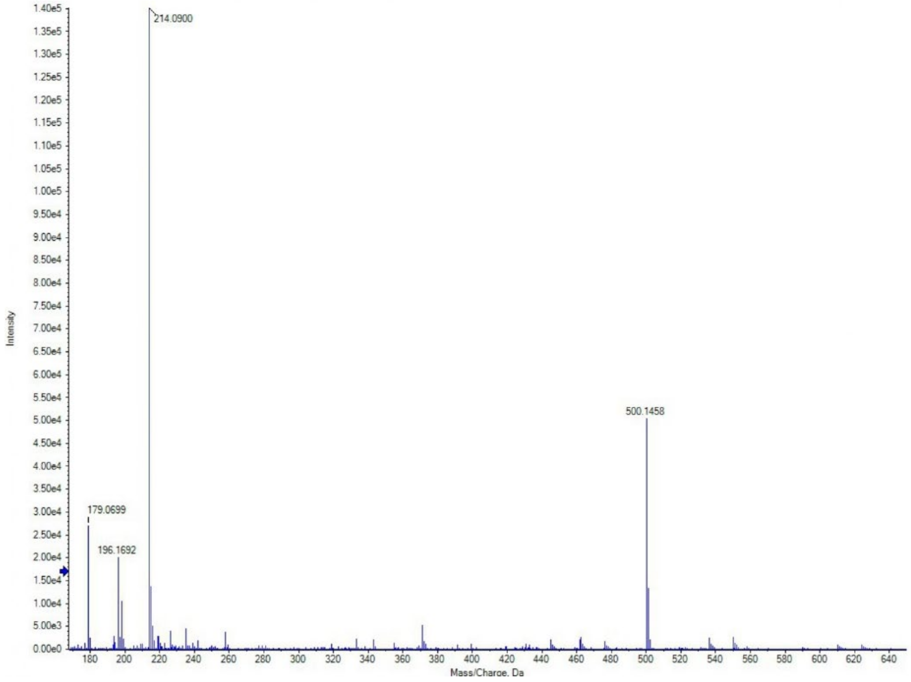

Figure S10. Mass spectroscopy data of compound **1**.

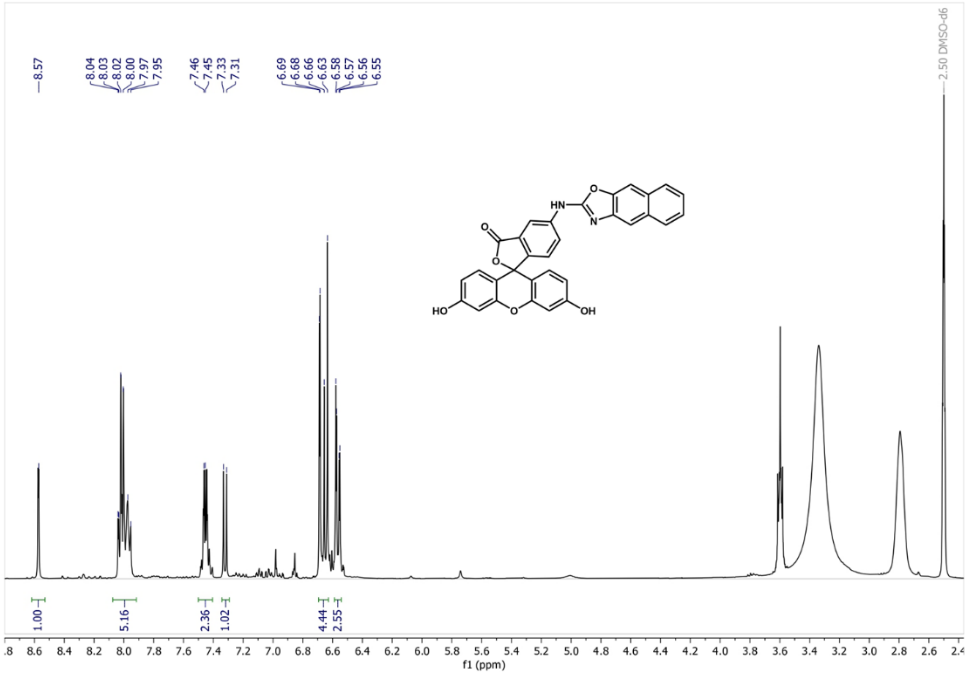

Figure S11. ^{1}H NMR spectrum of compound **9** in DMSO-d_6.

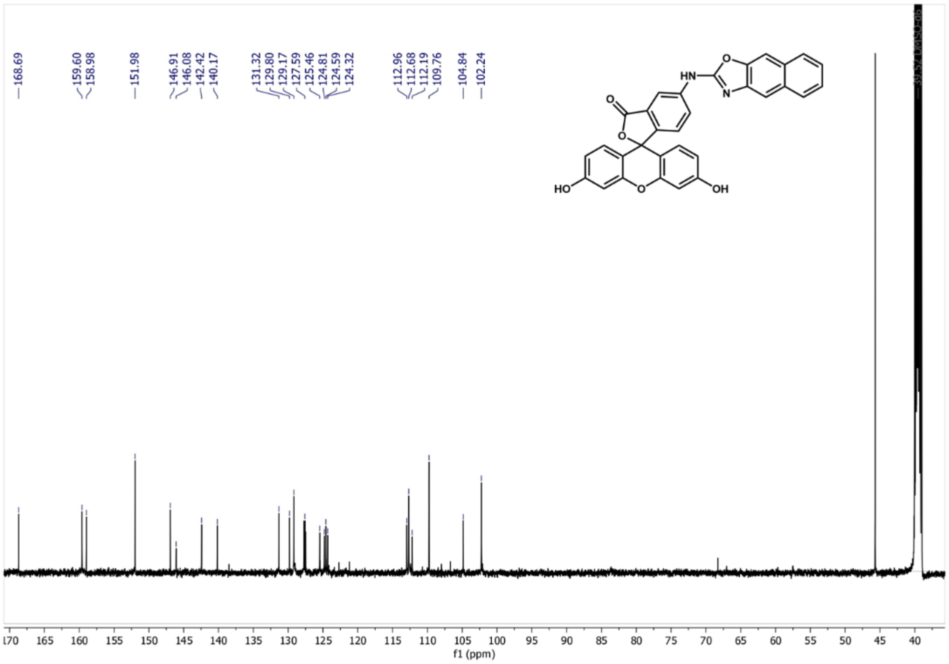

Figure S12. ^{13}C NMR spectrum of compound **9** in DMSO-d_6.

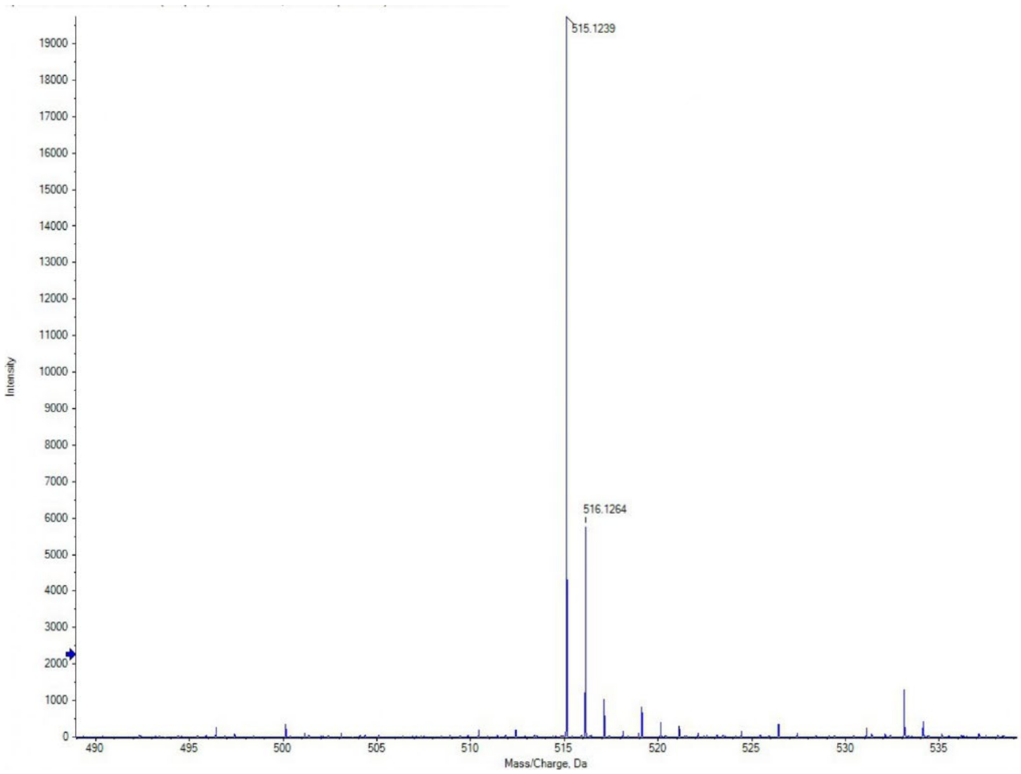

Figure S13. Mass spectroscopy data of compound **9**.

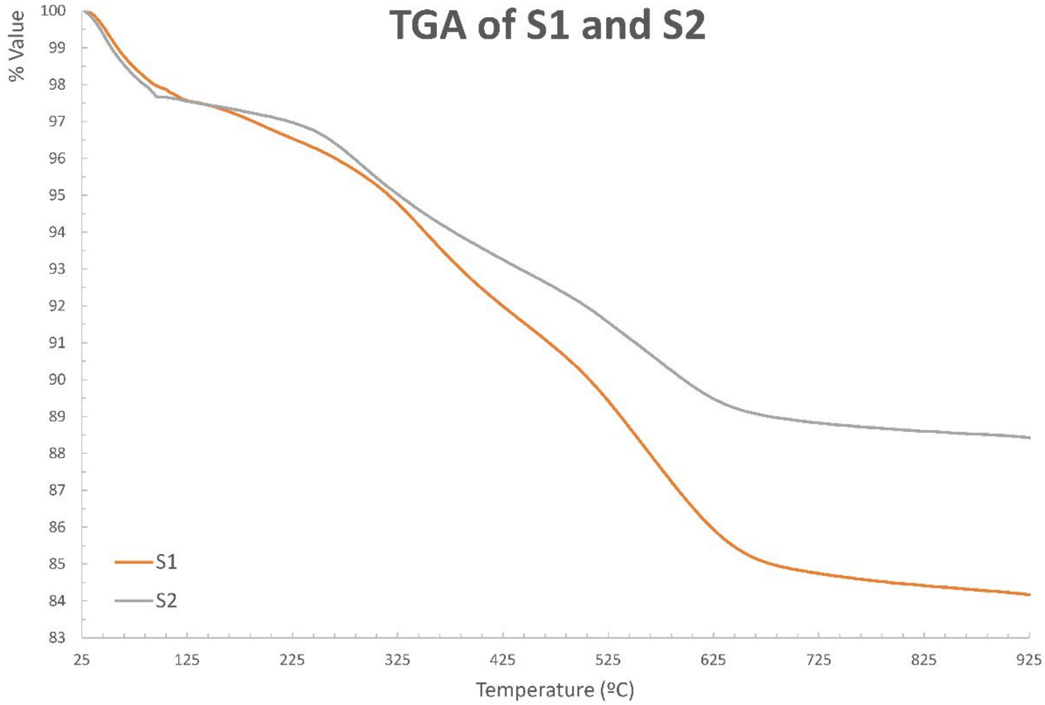

Figure S14. Thermogravimetric analysis (TGA) of solids **S1** and **S2.**

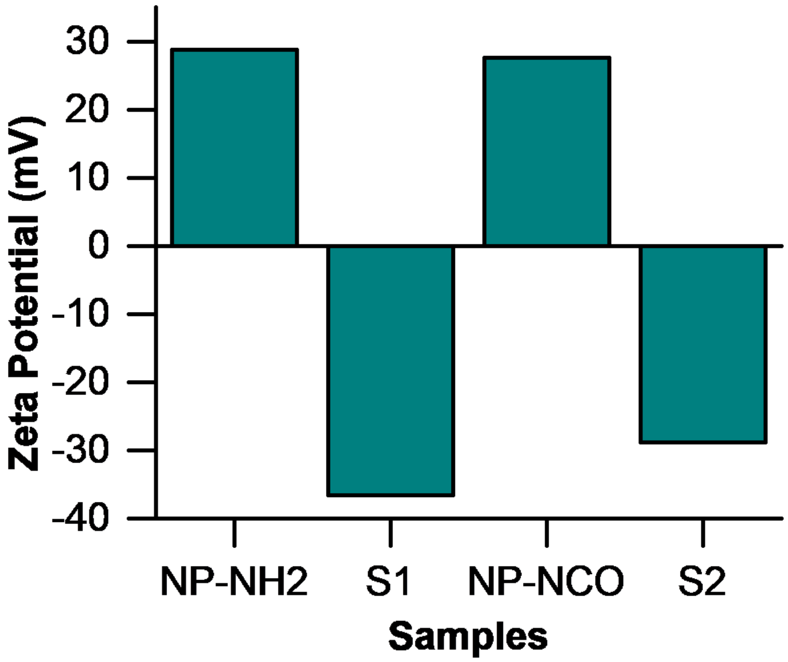

Figure S15. Zeta Potential for **SiNPs-APTES** (depicted as NP-NH2), **S1**, **SiNPs-NCO** (depicted as "NP-NCO") and **S2**.

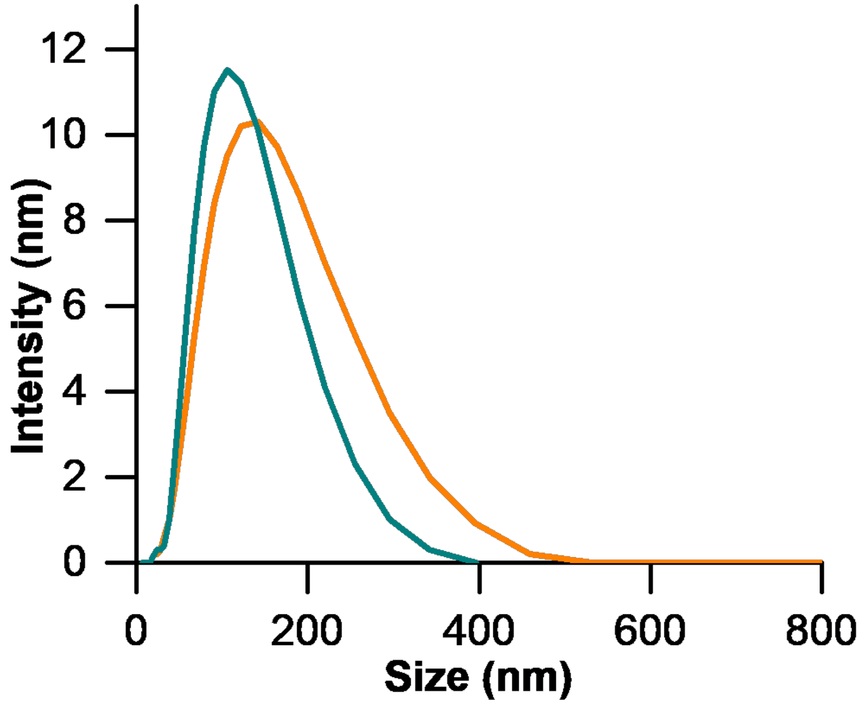

Figure S16. Hydrodynamic diameter distribution of **S1** (green line) and **S2** (orange line).

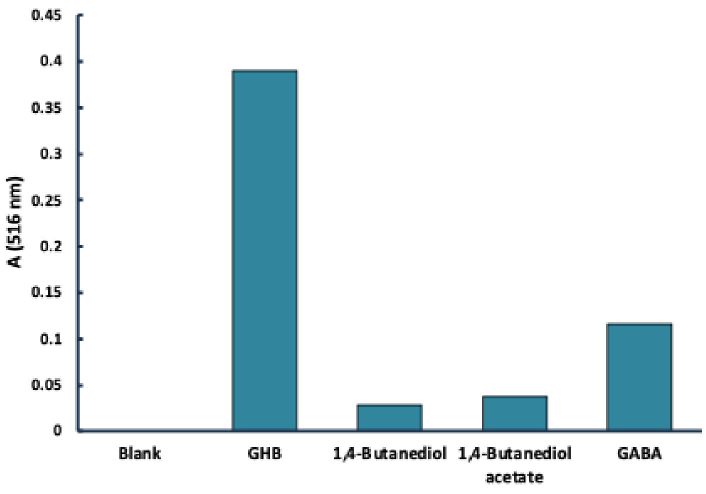

Figure S17. Absorbance at λ = 516 nm of nanosensor **S1** (0.33 mg/mL in DMSO) solutions containing GHB and different possible interferents (49.2 µM).

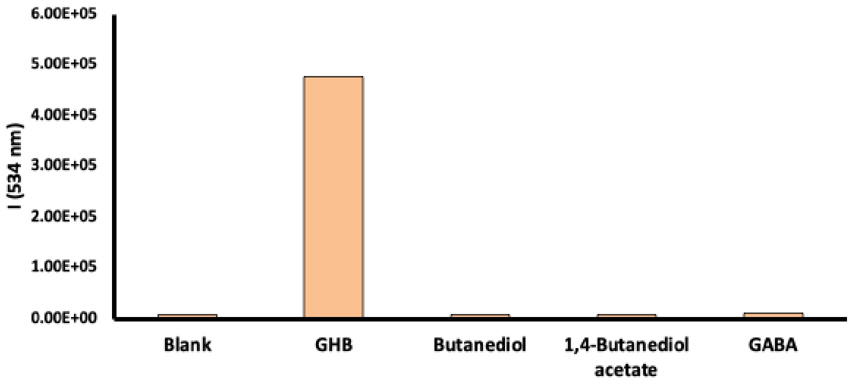

Figure S18. Emission intensity at λ = 534 nm (λ_{exc} = 490 nm) of nanosensor **S2** (1 mg/mL in DMSO) solutions containing GHB and different interferents (189 µM).

Table S1. Recovery and accuracy of the method with **S1** to detect GHB in aqueous samples were calculated according to reference K. A. Rawat, R. K. Singhal, S. K. Kailasa, RSC Adv. 6 (2016) 32025-32036. https://doi.org/10.1039/c6ra01575a

Analyte	Known concentration (µM)	Found concentration (µM)	Recovery %[a]	Accuracy %[b]
GHB	39	40.7	104%	4%

[a] recovery % (found concentration/known concentration) x 100
[b] accuracy % (found concentration – known concentration/known concentration) x 100

Table S2. Recovery and accuracy of the method with **S2** to detect GHB in aqueous samples were calculated according to reference K. A. Rawat, R. K. Singhal, S. K. Kailasa, RSC Adv. 6 (2016) 32025-32036. https://doi.org/10.1039/c6ra01575a

Analyte	Known concentration (µM)	Found concentration (µM)	Recovery %[a]	Accuracy %[b]
GHB	100	95.5	96%	-4%

[a] recovery % (found concentration/known concentration) x 100
[b] accuracy % (found concentration – known concentration/known concentration) x 100

Table S3. Comparison of the sensing properties of different optical selective probes for GHB detection.

Probe	Detection	LOD	Linear Range	Response Time	Ref.
BODIPY derivative	Fluorescence "Turn off" *In solution*	5 mg mL^{-1}	5 - 100 mg mL^{-1}	No data available	*Chem. Commun.*, **2014,** 50, 2904
Iridium (III) complex	Fluorescence "Turn off" *In solution*	0.15 mg mL^{-1}	0.15 – 1.3 mg mL^{-1}	No data available	*J. Mater. Chem. B*, **2017**, 5, 2739--2742
Naphthoxazole derivatives (**1** and **2**)	**1:** Colorimetric (yellow to red) **2:** Fluorescence "Turn on" *Both in solution*	**1:** 0.13 µM **2:** 0.12 µM	**1:** 0.2 - 8 µM **2:** 0.2 - 10 µM	Immediately after a basic pre-treatment of sample	*Chem. Commun.*, **2020,** 56, 12600-12603

Thiourea derivatives (**1** and **2**)	Colorimetric (Yellow to orange) *In solution*	**1:** 1.75 µM **2:** 0.77 µM	**1**: 2 - 12 µM **2:** 1 - 9 µM	Immediately after a basic pre-treatment of sample	*Analyst,* ***2021****, 146, 5601-5609*
PCDA-Gabazine	Colorimetric (blue to red) *In PCDA nanofibers*	9.6 µg mL^{-1}	0.015 – 0.5 µg μL^{-1}	No data available	*Sens. Actuators B: Chem.,* ***2021,*** *347, 130598*
Iron (III) complex	Colorimetric (red to yellow) Fluorescence “Turn on” *Both in solution*	0.25 µg mL^{-1} (abs.) 0.01 µg mL^{-1} (em.)	0.3 – 10 µg mL^{-1} (abs.) 0.05 – 10 µg mL^{-1} (em.)	Less than 5 seconds	*Sens. Actuators B: Chem.,* ***2022,*** *364, 131861*
Fluorene-containing cyanostilbenes	Fluorescence “Turn on” *In solution and chromatographic paper*	5 mM	5 - 50 mM	No data available	*J Photochem Photobiol A Chem.,* ***2022****, 427, 113844*
Copper (II) complex	Fluorescence “Turn on” *In glass fiber paper*	0.03 µM	0.02 – 0.5 µM	60 seconds (measured through smartphone)	*Sens. Actuators B: Chem.,* ***2023,*** *137, 133043*
Naphthoxazoles and benzoxazole derivatives	Fluorescence “Turn on” *In cellulose-based paper*	7.3 mM	10 - 125 mM	Immediately, but Maximum response after 1 hour	*Sens. BioSensing Res.,* ***2024****, 46, 100691*
Amino quinazolinone-based derivatives	Colorimetric (colorless to yellow) *In solution and LPAD paper*	0.012 mM (sol.) 4.13 mM (LPAD)	0.013 – 0.1 mM (sol.) 13.76 – 20 mM (LPAD)	Immediately	*Sens. Actuators B: Chem.,* ***2024,*** *418, 136228*
Naphthoxazole derivatives (**S1** and **S2** in silica nanoparticles)	**S1:** Colorimetric (yellow to red) **S2:** Fluorescence “Turn on” “Both *In suspension*” **1**: Colorimetric (pale pink to deep pink) “*In solution*”	**S1:** 2.21 µM **S2:** 1.65 µM **1:** 19.2 µM	**S1:** 3.7 -49.2 µM **S2:** 2.85-118.3 µM **1:** 32-132 µM	Immediately	*This work*
